# Environmentally Friendly Synthesis of Polysubstituted Pyrroles in Ionic Liquid via Gold-Catalyzed Propargylic Substitution/Hydration/Amination/Cycloisomerization Sequence

**DOI:** 10.3390/molecules31071203

**Published:** 2026-04-05

**Authors:** Hitomi Chiaki, Yukinori Umezawa, Yoshimitsu Hashimoto, Nobuyoshi Morita

**Affiliations:** Department of Pharmacy, Showa Pharmaceutical University, Machida 194-8543, Japan

**Keywords:** gold catalysts, ionic liquid, propargylic substitution, hydration, amination, cycloisomerization, pyrroles

## Abstract

An environmentally friendly synthesis of polysubstituted pyrroles in ionic liquid was achieved via a gold-catalyzed propargylic substitution/hydration/amination/cycloisomerization sequence. Treatment of propargylic alcohols, 1,3-dicarbonyl compounds, and arylamines in the presence of AuBr_3_ (5 mol%) and AgOTf (15 mol%) in [EMIM][NTf_2_] afforded polysubstituted pyrroles in good to high yield. This reaction involves reacting arylamine with the hydrated propargylic substitution product formed as an intermediate to yield polysubstituted pyrroles.

## 1. Introduction

The synthesis of cyclic compounds via sequential reaction is a potent and economical strategy for the synthesis of bioactive natural products and their derivatives [1,2,3,4,5], reducing both reaction time and waste generation. For example, gold catalysts have been employed in the synthesis of numerous cyclic compounds [6]. However, many of these sequential reactions are conducted in highly volatile organic solvents. Indeed, in sequential reactions using gold catalysts, highly toxic halogenated organic solvents are frequently employed. This is problematic because the Sustainable Development Goals (SDGs) promulgated by the United Nations require the development of sustainable chemical synthesis methods [7,8,9,10,11,12], and therefore developing organic reactions that minimize environmental impact is an urgent task [13,14,15,16,17,18].

One approach to developing sustainable chemical synthesis involves using ionic liquids instead of organic solvents [19,20]. Ionic liquids are salts that remain liquid below 100 degrees Celsius. The properties of these ionic liquids include (1) high thermal stability and a wide liquid temperature range, (2) negligible vapor pressure, (3) non-flammability and non-combustibility, (4) excellent dissolution properties for organometallic compounds, (5) controllability of melting point and solubility in organic solvents and water through selection of cation structures and anionic species in ionic liquid, (6) easy separation of reaction reagents (transition metal complexes, enzymes, etc.) and products, and (7) direct reusability of the ionic liquid containing the reagents (transition metal complexes, enzymes, etc.). For these reasons, ionic liquids are considered promising alternatives to organic solvents [21,22,23,24,25,26,27,28,29,30].

**Scheme 1:**
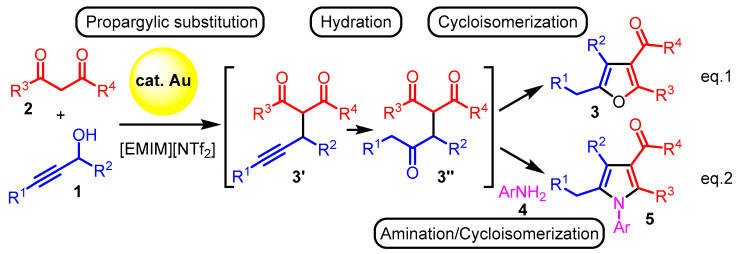
Gold-catalyzed reaction for the synthesis of polysubstituted furans **3** (Equation (1); previous work) and polysubstituted pyrroles **5** (Equation (2); this work).

We recently reported environmentally friendly and stereoselective syntheses of cyclic compounds (1,2,3-trisubstituted indanes [31,32] and 2,3-dihydrobenzofurans [33]) using reusable gold(I)/(III) catalysts in ionic liquids. Very recently, we have developed an environmentally benign synthesis of polysubstituted furans **3** in ionic liquid via a gold-catalyzed propargylic substitution/hydration/cycloisomerization sequence (Scheme 1, Equation (1): previous work) [34]. This reaction proceeds via the hydrated propargylic substitution product **3″**. We hypothesized that reaction of this hydrated propargylic substitution product **3″** with an amine **4** would result in amination followed by cycloisomerization, affording a polysubstituted pyrrole **5** (Scheme 1, Equation (2): This work). Several research groups have synthesized polysubstituted pyrroles [35,36] via similar sequential reactions starting from propargylic alcohol **1**, but all of them employ highly volatile and toxic halogenated organic solvents or toluene solvents [37,38,39,40]. The present research was initiated with the aim of expanding the application scope of our proprietary environmentally friendly gold-catalyzed reaction and achieving an unprecedented environmentally friendly pyrrole synthesis.

## 2. Results and Discussion

First, to synthesize polysubstituted pyrrole **5aaa** from propargylic alcohol **1a**, acetylacetone (**2a**), and *p*-methoxyaniline (**4a**), we optimized the reaction conditions for propargylic substitution/hydration/amination/cycloisomerization catalyzed by gold catalyst in an ionic liquid (Table 1). Our previous studies on furan synthesis confirmed that the propargylic substituted product **3**′**aa** forms after 10 min at room temperature, whereas the hydrated propargylic substituted product **3″aa** forms after 30 min at room temperature. The TLC behavior of these two products, **3**′**aa** and **3″aa**, has been analyzed [34]. Since the cycloisomerization of the hydrated propargylic substitution product **3″aa** to furan **3** requires a temperature of 60 °C, we predicted that the addition of *p*-methoxyaniline (**4a**) to the hydrated propargylic substitution product **3″aa** would cause amination and cycloisomerization to proceed preferentially, resulting in the conversion to polysubstituted pyrrole **5aaa**. Treatment of propargylic alcohol **1a** and acetylacetone (**2a**) with *p*-methoxyaniline (**4a**) in the presence of gold(I) catalyst (5 mol% AuCl) or gold(III) catalyst (5 mol% AuBr_3_) in ionic liquid [ethylmethylimidazolium (EMIM)][NTf_2_] at 100 °C for 1 day failed to give, or gave only a trace, of the desired product **5aaa** (entries 1 and 2), whereas addition of silver catalyst (5 mol% AgOTf for 5 mol% AuCl or 15 mol% AgOTf for 5 mol% AuBr_3_) to activate the gold catalyst afforded the desired pyrrole **5aaa** in 53% (entry 3) and 72% yields (entry 4), respectively. Lowering the reaction temperature to 60 °C and extending the reaction time by three days improved the yield (entry 5), while reducing the amount of gold and silver catalysts decreased the yield (entry 6). The reaction with AgOTf (15 mol%) at 60 °C for 3 days afforded trace amounts of pyrroles **5aaa** (entry 7).

Next, we examined the effect of the anion part of ionic liquid (Table 2). The reaction with [EMIM][OTf], [EMIM][HSO_4_], [EMIM][MeSO_4_], [EMIM][Me_2_PO_4_], and [EMIM][CH_3_CO_2_] did not proceed at all (entries 2–6), whereas the reaction with [EMIM][NTf_2_] afforded the desired product **5aaa** in high yield (entry 1). In the case of organic solvents (toluene, ClCH_2_CH_2_Cl, THF), the reaction gave a low yield of pyrrole **5aaa** or no product (entries 7–9).

**Figure 1:**
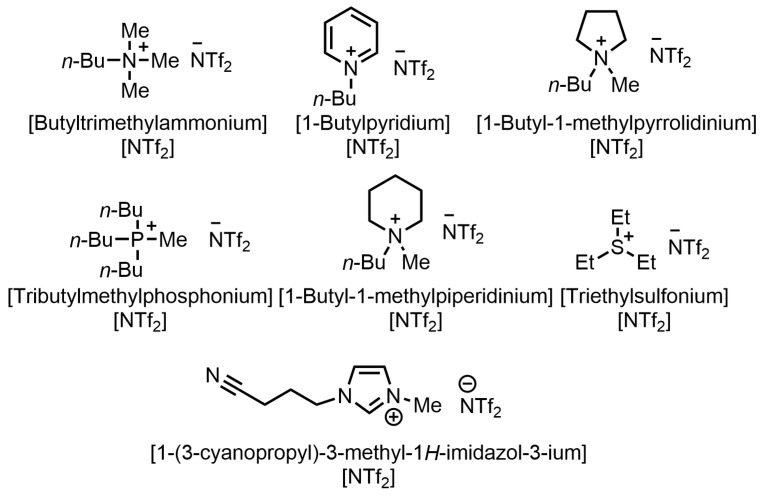
Ionic liquids with different cation groups.

We also investigated the effect of cation part of ionic liquid (Table 3, Figure 1). In the reactions with [butyltrimethylammonium][NTf_2_], [1-butylpyridium][NTf_2_], [1-butyl-1-methylpyrrolidinium][NTf_2_], [tributylmethylphophonium][NTf_2_], and [1-butyl-1-methylpiperidinium][NTf_2_], pyrrole **5aaa** was obtained in good to high yields (entries 2–6), whereas in the reaction with [triethylsulfonium][NTf_2_] and [1-(3-cyanopropyl)-3-methyl-1*H*-imidazol-3-ium][NTf_2_], no product was obtained at all (entries 7, 8). These results indicate that pyrrole **5aaa** is obtained in high yield in the reaction using ionic liquid [EMIM][NTf_2_] (entry 1).

**Scheme 2:**
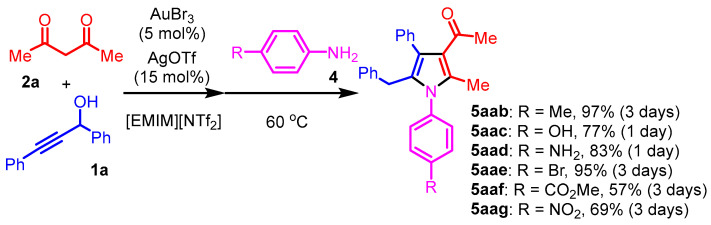
Scope and limitations of gold-catalyzed reactions with various arylamines **4** for the synthesis of polysubstituted pyrroles **5aab**–**5aag**.

We next conducted the gold-catalyzed reactions with various types of arylamines **4b**–**g** bearing substituents at the *p*-position (Scheme 2). Treatment of propargylic alcohol **1a** and acetylacetone (**2a**) with arylamines **4b**–**d** bearing electron-rich substituents in the presence of AuBr_3_ (5 mol%) and AgOTf (15 mol%) in ionic liquid [EMIM][NTf_2_] afforded the corresponding polysubstituted pyrroles **5aab**–**5aad** in good to high yields. Furthermore, when the substituent on the aromatic ring of the arylamine **4** was a hydroxyl group or an amino group, the reaction time was shortened and the reaction was completed within one day. In the case of an electron-withdrawing group (**4e**; R = Br, **4f**; R = CO_2_Me), the gold-catalyzed reaction with arylamine **4e** having a bromine group furnished polysubstituted pyrrole **5aae** in high yield, whereas the reaction with arylamine **4f** bearing an ester group gave a moderate yield of the corresponding pyrrole **5aaf**. Even in the case of an aniline **4g** containing a nitro group which had been concerned the coordination to the gold center, the reaction proceeded smoothly to yield the corresponding pyrrole **5aag** in good yield.

**Scheme 3:**
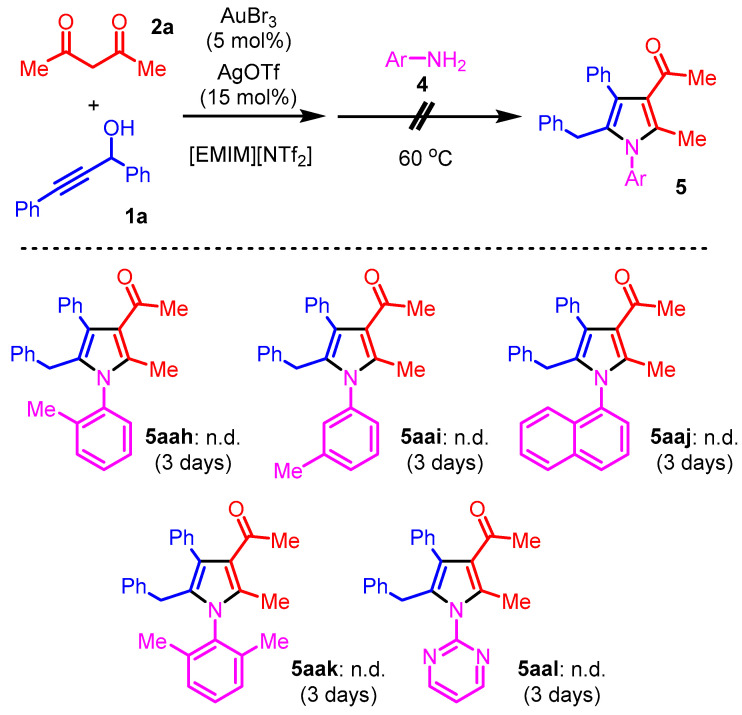
Scope and limitations of the gold-catalyzed reactions with arylamines **4g**–**l**.

Next, we investigated the reactions with propargylic alcohol **1a**, acetylacetone (**2a**) and various types of arylamines, **4h**–**k** or 2-amino-pyrimidine (**4l**) (Scheme 3). Unfortunately, the reactions with arylamines **4h**–**l** did not proceed, probably due to steric hindrance in the cases of **4h**–**k**, and the basicity of the nitrogen atoms in **4l**.

**Scheme 4:**
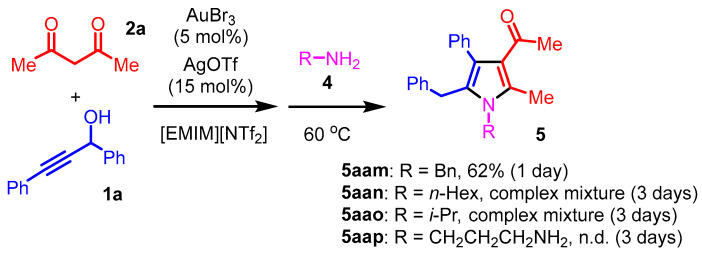
Scope and limitations of the gold-catalyzed reactions with alkylamines **4m**–**p**.

We also investigated the gold-catalyzed reactions with alkylamines **4m**–**p** instead of arylamine (Scheme 4). The reaction with benzylamine **4m** afforded the corresponding pyrrole **5aam** in moderate yield, whereas the reaction with *n*-hexylamine **4n** or *i*-propylamine **4o** resulted in a complex mixture. In the case of 1,3-diamine **4p**, no product was obtained.

**Scheme 5:**
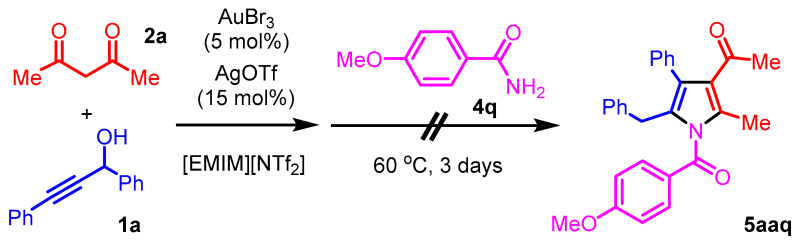
The gold-catalyzed reaction with 4-methoxybenzamide (**4q**).

Although we examined the gold-catalyzed reaction with 4-methoxybenzamide (**4q**) instead of arylamine, the desired product **5aaq** was not formed (Scheme 5).

**Scheme 6:**
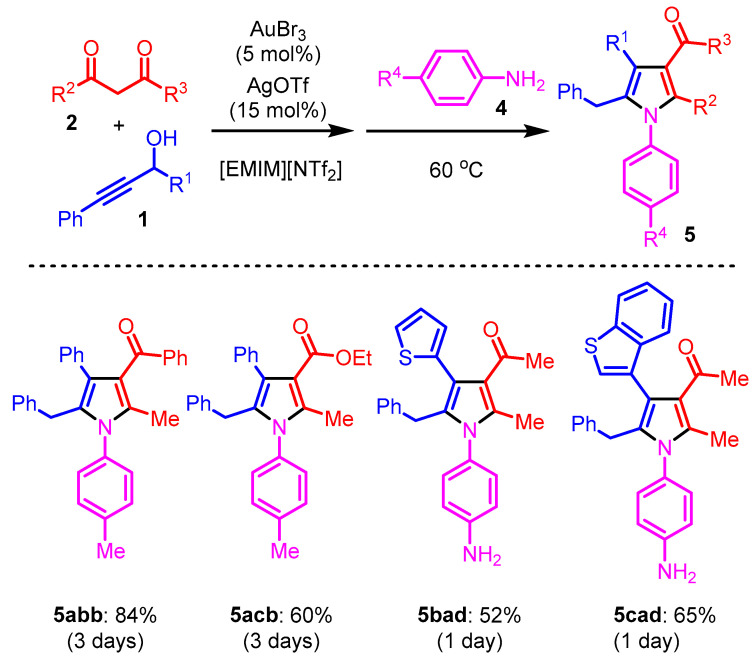
Gold-catalyzed propargylic substitution/hydration/amination/cycloisomerization sequence for the synthesis of polysubstituted pyrroles **5**.

Next, we examined the gold-catalyzed reactions with different starting materials **1b**–**c** and active methylene compounds **2b**–**c** instead of different propargylic alcohols or acetylacetone (**2a**) (Scheme 6). Treatment of propargylic alcohol **1a** and active methylene compounds **2b**–**c** with *p*-methylaniline (**3b**) afforded the corresponding pyrroles **5abb**–**5acb** in moderate to good yields. The gold-catalyzed reaction with propargylic alcohol bearing thiophene **1b** or benzothiophene **1c** at the propargylic position afforded moderate yields of the corresponding pyrroles **5bad**–**5cad**.

**Scheme 7:**
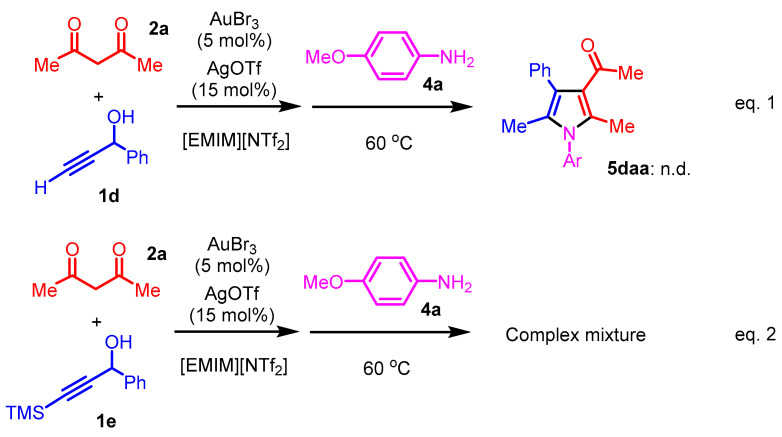
The gold-catalyzed reactions with propargylic alcohols **1d**–**e**.

In addition, different types of propargylic alcohols **1d**–**e** were examined in the reactions (Scheme 7). Unfortunately, no desired product was obtained.

**Scheme 8:**
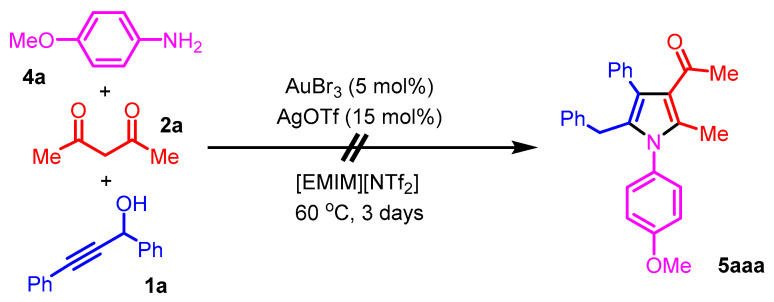
The gold-catalyzed three-component reaction.

We further examined the gold-catalyzed three-component reaction of propargylic alcohol **1a**, acetylacetone (**2a**) and *p*-methoxyaniline (**4a**) (Scheme 8). However, this reaction did not proceed at all.

**Scheme 9:**
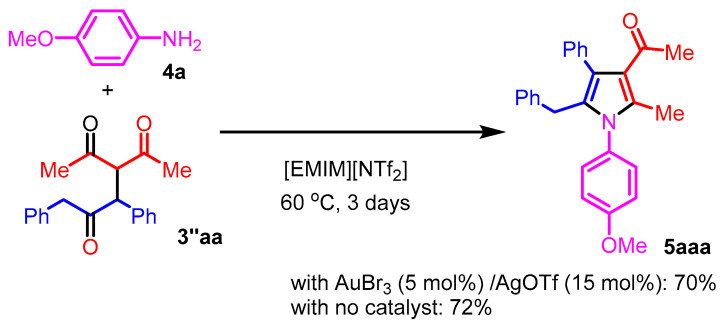
The gold-catalyzed reaction from hydrated propargylic substitution product **3″aa** to pyrrole **5aaa**.

To confirm the reaction pathway, the gold-catalyzed reaction from hydrated propargylic substitution product **3″aa** [34] was carried out (Scheme 9). Treatment of hydrated propargylic substitution product **3″aa** with *p*-methoxyaniline (**4a**) in the presence of AuBr_3_ (5 mol%) and AgOTf (15 mol%) in ionic liquid [EMIM][NTf_2_] furnished the desired pyrrole **5aaa** in 70% yield. Furthermore, since the reaction proceeded smoothly even without gold catalyst and pyrrole **5aaa** was produced in good yield, it would be presumed that the gold catalyst does not involve the amination/cycloisomerization steps (Scheme 9).

**Scheme 10:**
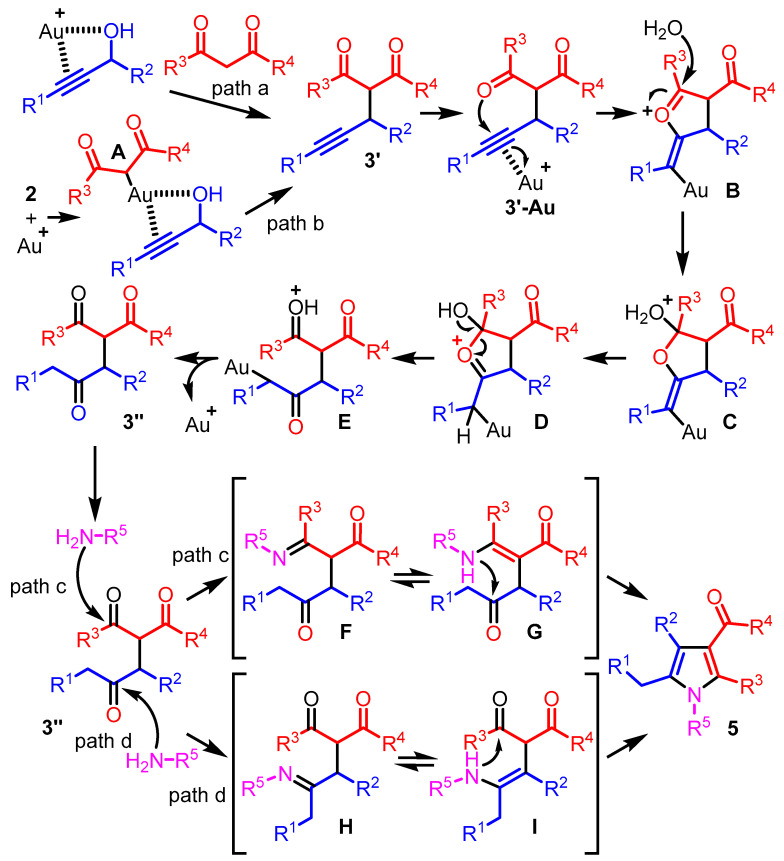
Plausible reaction mechanism of the gold-catalyzed propargylic substitution/hydration/amination/cycloisomerization sequence for the synthesis of polysubstituted pyrrole **5**.

A plausible reaction mechanism of the gold-catalyzed propargylic substitution/hydration/amination/cycloisomerization sequence in ionic liquid [EMIM][NTf_2_] for the synthesis of polysubstituted pyrroles **5** is shown in Scheme 10. Regarding the first-stage propargylic substitution that yields the propargyl substitution product **3′**, two possibilities can be considered. The first possibility is that the gold catalyst activates propargylic alcohol **1** by coordinating with the oxygen atom of the hydroxyl group and the triple bond in propargylic alcohol **1**, thereby initiating the propargylic substitution with active methylene compound **2** (Scheme 10, path a). The second possibility is that the gold complex **A** [41,42,43,44,45,46,47] formed by reaction between the gold catalyst and the active methylene compound **2** undergoes the propargylic substitution while maintaining a coordination structure **A** similar to that in the case of the first possibility (Scheme 10, path b). Next, activation of the triple bond in the propargylic substitution product **3′** by the gold catalyst promotes the cyclization reaction of the carbonyl group, forming a vinyl gold complex **B** (**3**′→**3**′**-Au**→**B**). Water attacks the vinyl gold complex **B**, triggering an opening reaction to form a hydrated propargylic substitution product **3″** (**B**→**C**→**D**→**E**→**3″**). The formation of pyrrole **5** via amination from the hydrated propargylic substitution product **3″** in the final stage can be explained in two ways. The first possibility is that amination occurs at the carbonyl group of the hydrated propargylic substitution product **3″**, followed by cyclization from the imino nitrogen and subsequent isomerization to yield pyrrole **5** (Scheme 10, pathway c; **3″**→**F**→**G**→**5**). The second possibility is that amination proceeds at the newly formed carbonyl group of the hydrated propargylic substitution product **3″**, followed by cyclization from the imino nitrogen to form pyrrole **5** (Scheme 10, pathway d; **3″**→**H**→**I**→**5**).

Finally, we investigated the reusability of the gold catalyst in ionic liquid. Although we attempted various approaches, unfortunately, reuse was unsuccessful. The reason for the inability to reuse would be presumed to be due to deactivation caused by gold nanoparticles generated through the reaction between the gold catalyst and amines **4** [48,49]. Further investigation is in progress.

## 3. Materials and Methods

### 3.1. General Information

NMR spectroscopic investigations were carried out on a JEOL JNM-ECZ400S (Japan Electron Optics Laboratory Co., Ltd., Tokyo, Japan) or Bruker AV-400NEO spectrometer (Bruker, Billerica, MA, USA) at room temperature, with tetramethylsilane as an internal standard (CDCl_3_ solution). The chemical shift δ is given in parts per million (ppm), the coupling constant *J* in Hertz (Hz). The calibration was performed on the residual protons of the deuterated solvent used, in this case deuterated chloroform (CDCl_3_:δ (1H) = 7.27, δ (13C) = 77.0). Infrared spectra (IR) were measured on an IR spectrometer (Shimadzu IRSpirit; Shimadzu Corporation, Kyoto, Japan) and are reported in wavenumbers (cm^−1^). Mass spectra were obtained with a JEOL JMS-700 spectrometers (Japan Electron Optics Laboratory Co., Ltd., Tokyo, Japan). Column chromatography and thin layer chromatography (TLC) were carried out with Merck silica gel 60 (1.09385) (Merck, Darmstadt, Germany) and Merck silica gel 60 F254 (Merck, Darmstadt, Germany).

### 3.2. General Procedure of Gold(III)-Catalyzed Propargylic Substitution/Hydration/Amination/Cycloisomerization for the Synthesis of Polysubstituted Pyrroles ***5*** from Propargylic Alcohols ***1***

At room temperature, a solution of propargylic alcohol **1** and 1,3-dicarbonyl compound **2** in [ethylmethylimidazolium (EMIM)][NTf_2_] (1 mL) was treated with 5 mol% AuBr_3_ and 15 mol% AgOTf. The reaction mixture was stirred at 60 °C. After complete consumption of propargylic alcohol **1**, the formation of the hydrated propargylic substitution product **3″** was confirmed by thin-layer chromatography (TLC). Amine **4** was added to the reaction mixture, and stirring was continued at 60 °C for 1 to 3 days. After adding diethyl ether (5 mL × 5), the organic layer was extracted, collected, washed with brine, and dried over Na_2_SO_4_. The solvent was removed under reduced pressure. The crude product was subjected to SiO_2_ column chromatography (hexane/AcOEt = 20:1) to afford polysubstituted pyrrole **5**.

*1-[5-Benzyl-1-(4-methoxyphenyl)-2-methyl-4-phenyl-1H-pyrrol-3-yl]ethan-1-one* (**5aaa**): Propargylic alcohol **1a** (31 mg, 0.15 mmol, acetylacetone (**2a**) (15 mg, 0.15 mmol), *p*-methoxyaniline (**4a**) (36 mg, 0.29 mmol), AuBr_3_ (3.3 mg, 0.007 mmol, 5 mol%) and AgOTf (5.8 mg, 0.022 mmol, 15 mol%) in [EMIM][NTf_2_] (1 mL) furnished pyrrole **5aaa** (54 mg, yield = 93%) as a colorless solid; Mp 153.0–153.6 °C; IR (ATR) 3027, 2923, 1649, 1605, 1511, 1249 cm^–1^; ^1^H NMR (400 MHz, CDCl_3_) δ 7.38 (4H, d, *J* = 4.5 Hz), 7.33–7.28 (1H, m), 7.06–7.02 (3H, m), 6.85–6.77 (4H, m), 6.65–6.62 (2H, m), 3.81 (3H, s), 3.64 (2H, s), 2.24 (3H, s), 1.95 (3H, s); ^13^C NMR (100 MHz, CDCl_3_) δ 197.4, 159.4, 139.6, 136.7, 136.2, 130.6, 129.8, 129.7, 129.4, 128.3, 128.0, 127.9, 126.8, 125.7, 123.5, 121.6, 114.1, 55.5, 31.0, 30.7, 12.9; HRMS (EI) m/z calcd for C_27_H_25_NO_2_ [M]^+^ 395.1885, found 395.1887.
*1-[5-Benzyl-2-methyl-4-phenyl-1-(p-tolyl)-1H-pyrrol-3-yl]ethan-1-one* (**5aab**): Propargylic alcohol **1a** (35 mg, 0.16 mmol), acetylacetone (**2a**) (16 mg, 0.16 mmol), *p*-toluidine (**4b**) (34 mg, 0.31 mmol), AuBr_3_ (3.4 mg, 0.008 mmol, 5 mol%) and AgOTf (6.1 mg, 0.024 mmol, 15 mol%) in [EMIM][NTf_2_] (1 mL) furnished pyrrole **5aab** (62 mg, yield = 97%) as a colorless solid as a white solid; Mp 134.3–134.9 °C; IR (ATR) 3029, 2923, 1650,1515,1410 cm^–1^; ^1^H-NMR (400 MHz, CDCl_3_) δ 7.37–7.28 (5H, m), 7.09 (2H, d, *J* = 8.0 Hz), 7.04–7.01 (3H, m), 6.81 (2H, d, *J* = 8.0 Hz), 6.64–6.61 (2H, m), 3.65 (2H, s), 2.36 (3H, s), 2.24 (3H, s), 1.95 (3H, s); ^13^C-NMR (100 MHz, CDCl_3_) δ 197.5, 139.7, 138.5, 136.8, 136.1, 134.6, 130.7, 129.6, 129.5, 128.4, 128.2, 128.1, 127.9, 126.9, 125.8, 123.7, 121.8, 31.1, 30.8, 21.2, 13.0; HRMS (EI) m/z calcd for C_27_H_25_NO [M]^+^ 379.1936, found 379.1937.
*1-[5-Benzyl-1-(4-hydroxyphenyl)-2-methyl-4-phenyl-1H-pyrrol-3-yl]ethan-1-one* (**5aac**): Propargylic alcohol **1a** (30 mg, 0.14 mmol), acetylacetone (**2a**) (15 mg, 0.14 mmol), 4-aminophenol (**4c**) (32 mg, 0.29 mmol), AuBr_3_ (3.2 mg, 0.007 mmol, 5 mol%) and AgOTf (5.6 mg, 0.022 mmol, 15 mol%) in [EMIM][NTf_2_] (1 mL) furnished pyrrole **5aac** (42 mg, yield = 77%) as a colorless oil; IR (ATR) 3210, 2923, 2854, 1625, 1602, 1516, 1454 cm^–1^; ^1^H NMR (400 MHz, CDCl_3_) δ 7.38 (4H, d, *J* = 4.3 Hz), 7.34–7.28 (1H, m), 7.07–7.01 (3H, m), 6.75 (4H, s), 6.65–6.61 (2H, m), 3.64 (2H, s), 2.25 (3H, s), 1.97 (3H, s); ^13^C NMR (100 MHz, CDCl_3_) δ 198.1, 156.3, 139.5, 136.9, 136.6, 130.6, 130.0, 129.5, 129.3, 128.4, 128.0, 127.9, 126.9, 125.7, 123.6, 121.3, 115.7, 30.9, 30.7, 13.1; HRMS (EI) m/z calcd for C_26_H_23_NO_2_ [M]^+^ 381.1729, found 381.1725.
*1-[1-(4-Aminophenyl)-5-benzyl-2-methyl-4-phenyl-1H-pyrrol-3-yl]ethan-1-one* (**5aad**): Propargylic alcohol **1a** (30 mg, 0.14 mmol), acetylacetone (**2a**) (15 mg, 0.14 mmol), 1,4-phenylenediamine (**4d**) (32 mg, 0.29 mmol), AuBr_3_ (3.3 mg, 0.007 mmol, 5 mol%) and AgOTf (5.7 mg, 0.022 mmol, 15 mol%) in [EMIM][NTf_2_] (1 mL) furnished pyrrole **5aad** (46 mg, yield = 83%) as a colorless oil; IR (ATR) 3462, 3358, 3225, 3027, 2923, 1636, 1603, 1511, 1411 cm^–1^; ^1^H-NMR (300 MHz, CDCl_3_) δ 7.37–7.36 (4H, d, *J* = 4.4 Hz), 7.32–7.27 (1H, m), 7.07–7.02 (3H, m), 6.69 (2H, d, *J* = 8.8 Hz), 6.66 (2H, dd, *J* = 8.0, 2.8 Hz), 6.58 (2H, d, *J* = 8.8 Hz), 3.65 (2H, s), 2.24 (3H, s), 1.94 (3H, s); ^13^C-NMR (75 MHz, CDCl_3_) δ 197.3, 139.8, 136.9, 136.4, 130.6, 130.0, 129.2, 128.3, 128.1, 127.9, 126.8, 125.7, 123.4, 121.5, 115.2, 31.0, 30.7, 13.0 (several signals overlapped); HRMS (EI) m/z calcd for C_26_H_24_N_2_O [M]^+^ 380.1889, found 380.1877.
*1-[5-Benzyl-1-(4-bromophenyl)-2-methyl-4-phenyl-1H-pyrrol-3-yl]ethan-1-one* (**5aae**): Propargylic alcohol **1a** (30 mg, 0.14 mmol), acetylacetone (**2a**) (15 mg, 0.14 mmol), 4-bromoaniline (**4e**) (50 mg, 0.29 mmol), AuBr_3_ (3.2 mg, 0.007 mmol, 5 mol%) and AgOTf (5.9 mg, 0.022 mmol, 15 mol%) in [EMIM][NTf_2_] (1 mL) furnished pyrrole **5aae** (37 mg, yield = 95%) as a colorless oil; IR (ATR) 3060, 3029, 2923, 1650, 1603, 1511, 1491, 1408 cm^−1^; ^1^H-NMR (300 MHz, CDCl_3_) δ 7.42–7.38 (6H, m), 7.38–7.30 (1H, m), 7.30–7.06 (3H, m), 6.78 (2H, dd, *J* = 8.4, 2.0 Hz), 6.65–6.62 (2H, m), 3.64 (2H, s), 2.23 (3H, s), 1.94 (3H, s); ^13^C-NMR (75 MHz, CDCl_3_) δ 197.3, 139.2, 136.3, 136.2, 135.7, 132.2, 130.5, 130.0, 129.3, 128.4, 128.1, 128.0, 127.0, 125.9, 124.0, 122.6, 122.0, 31.0, 30.7, 12.9; HRMS (EI) m/z calcd for C_26_H_22_BrNO [M]^+^ 443.0885, found 443.0881.
*Methyl 4-(3-acetyl-5-benzyl-2-methyl-4-phenyl-1H-pyrrol-1-yl)benzoate* (**5aaf**): Propargylic alcohol **1a** (30 mg, 0.14 mmol), acetylacetone (**2a**) (14 mg, 0.14 mmol), methyl 4-aminobenzoate (**4f**) (43 mg, 0.28 mmol), AuBr_3_ (3.1 mg, 0.007 mmol, 5 mol%) and AgOTf (5.5 mg, 0.021 mmol, 15 mol%) in [EMIM][NTf_2_] (1 mL) furnished pyrrole **5aaf** (34 mg, yield = 57%) as a colorless oil; IR (ATR) 3027, 2950, 1723, 1652, 1603, 1509, 1435, 1407 cm^−1^; ^1^H NMR (400 MHz, CDCl_3_) δ 7.97 (2H, dd, *J* = 8.4, 2.0 Hz), 7.34–7.39 (4H, m), 7.34–7.31 (1H, m), 7.03–6.99 (5H, m), 6.60–6.57 (2H, m), 3.94 (3H, s), 3.67 (2H, s), 2.24 (3H, s), 1.95 (3H, s); ^13^C NMR (100 MHz, CDCl_3_) δ 197.4, 166.2, 141.3, 139.0, 136.3, 135.6, 130.5, 130.4, 130.2, 129.2, 128.5, 128.4, 128.0, 127.9, 127.1, 125.9, 124.3, 122.2, 52.4, 31.0, 30.7, 12.9; HRMS (EI) m/z calcd for C_28_H_25_O_3_N [M]^+^ 423.1834, found 423.1825.
*1-(5-Benzyl-2-methyl-1-(4-nitrophenyl)-4-phenyl-1H-pyrrol-3-yl)ethan-1-one* (**5aag**): Propargylic alcohol **1a** (35 mg, 0.17 mmol), acetylacetone (**2a**) (17 mg, 0.17 mmol), 4-nitroaniline (**4g**) (46 mg, 0.33 mmol), AuBr_3_ (3.6 mg, 0.008 mmol, 5 mol%) and AgOTf (6.4 mg, 0.025 mmol, 15 mol%) in [EMIM][NTf_2_] (1 mL) furnished pyrrole **5aag** (47 mg, yield = 69%) as a colorless oil; IR (ATR) 3029, 2992, 1717, 1651, 1597, 1559, 1527, 1496, 1455, 1406 cm^−1^; ^1^H NMR (400 MHz, CDCl_3_) δ 8.15 (2H, dd, *J* = 8.4, 2.0 Hz), 7.42–7.38 (4H, m), 7.36–7.32 (1H, m), 7.10 (2H, dd, *J* = 8.4, 2.0 Hz), 7.05–7.02 (3H, m), 6.61–6.59 (2H, m), 3.68 (2H, s), 2.25 (3H, s), 1.96 (3H, s); ^13^C NMR (100 MHz, CDCl_3_) δ 197.3, 147.3, 143.0, 138.7, 135.9, 135.3, 130.4, 129.4, 129.0, 128.5, 128.2, 127.8, 127.3, 126.2, 124.8, 124.3, 122.6, 31.0, 30.7, 12.9; HRMS (EI) m/z calcd for C_26_H_22_ N_2_O_3_ [M]^+^ 410.1630, found 410.1628.
*1-(1,5-Dibenzyl-2-methyl-4-phenyl-1H-pyrrol-3-yl)ethan-1-one* (**5aam**): Propargylic alcohol **1a** (29 mg, 0.14 mmol), acetylacetone (**2a**) (14 mg, 0.14 mmol), benzylamine (**4m**) (29 mg, 0.27 mmol), AuBr_3_ (3.0 mg, 0.007 mmol, 5 mol%) and AgOTf (5.3 mg, 0.021 mmol, 15 mol%) in [EMIM][NTf_2_] (1 mL) furnished pyrrole **5aam** (32 mg, yield = 62%) as a colorless oil; IR (ATR) 3029, 2920, 1647, 1603, 1514, 1494, 1454, 1407 cm^−1^; ^1^H-NMR (300 MHz, CDCl_3_) δ 7.35–7.21 (10H, m), 7.07 (1H, d, *J* = 7.2 Hz), 6.99 (2H, d, *J* = 7.2 Hz), 6.85 (2H, d, *J* = 7.2 Hz), 4.86 (2H, s), 3.72 (2H, s), 2.46 (3H, s), 1.94 (3H, s); ^13^C-NMR (75 MHz, CDCl_3_) δ 197.4, 139.3, 136.9, 136.8, 135.3, 130.5, 128.9, 128.6, 128.3, 127.9, 127.8, 127.4, 126.9, 126.3, 125.6, 124.5, 121.8, 31.1, 30.3, 11.8 (one signal overlapped); HRMS (EI) m/z calcd for C_27_H_25_NO [M]^+^ 379.1936, found 379.1945.
*(5-Benzyl-2-methyl-4-phenyl-1-(p-tolyl)-1H-pyrrol-3-yl)(phenyl)methanone* (**5abb**): Propargylic alcohol **1a** (31 mg, 0.15 mmol), 1-phenyl-1,3-butanedione (**2b**) (24 mg, 0.15 mmol), *p*-toluidine (**4b**) (32 mg, 0.30 mmol), AuBr_3_ (3.3 mg, 0.007 mmol, 5 mol%) and AgOTf (5.8 mg, 0.022 mmol, 15 mol%) in [EMIM][NTf_2_] (1 mL) furnished pyrrole **5abb** (55 mg, yield = 84%) as a white solid; Mp 146.0–146.5 °C; IR (ATR) 3027, 2920, 1633, 1599, 1578, 1515, 1447, 1408 cm^−1^; ^1^H NMR (400 MHz, CDCl_3_) δ 7.69–7.67 (2H, m), 7.28–7.23 (2H, m), 7.17–7.10 (5H, m), 7.10–7.02 (6H, m), 6.79 (2H, dt, *J* = 2.5, 2.5 Hz), 6.64–6.62 (2H, m), 3.64 (2H, s), 2.23 (3H, s), 1.94 (3H, s); ^13^C-NMR (100 MHz, CDCl_3_) δ 197.4, 166.2, 141.3, 139.0, 136.3, 135.6, 130.5, 130.4, 130.2, 129.2, 128.5, 128.4, 128.0, 127.9, 127.1, 125.9, 124.3, 122.2, 52.4, 31.0, 30.7, 12.9 (several signals overlapped); HRMS (EI) m/z calcd for C_32_H_27_NO [M]^+^ 441.2093, found 441.2082.
*Ethyl 5-benzyl-2-methyl-4-phenyl-1-(p-tolyl)-1H-pyrrole-3-carboxylate* (**5acb**): Propargylic alcohol **1a** (32 mg, 0.15 mmol), ethyl acetoacetate (**2c**) (20 mg, 0.15 mmol), *p*-toluidine (**4b**) (33 mg, 0.30 mmol), AuBr_3_ (3.4 mg, 0.008 mmol, 5 mol%) and AgOTf (6.0 mg, 0.023 mmol, 15 mol%) in [EMIM][NTf_2_] (1 mL) furnished pyrrole **5acb** (37 mg, yield = 60%) as a colorless oil; IR (ATR) 3030, 2979, 2925, 1693, 1603, 1516, 1494, 1454, 1415 cm^−1^; ^1^H NMR (400 MHz, CDCl_3_) δ 7.37–7.29 (4H, m), 7.26–7.22 (1H, m), 7.08 (2H, d, *J* = 8.0), 7.05–7.01 (3H, m), 6.80 (2H, dt, *J* = 8.3, 2.1 Hz), 6.66–6.64 (2H, m), 4.09 (2H, q, *J* = 7.2 Hz), 3.68 (2H, s), 2.36 (3H, s), 2.27 (3H, s), 1.02 (3H, t, *J* = 7.1 Hz); ^13^C NMR (100 MHz, CDCl_3_) δ 166.0, 139.8, 138.3, 136.6, 136.4, 134.7, 130.4, 129.5, 129.4, 128.2, 128.1, 127.8, 127.5, 126.1, 125.6, 124.0, 111.0, 59.2, 30.7, 21.2, 13.9, 12.5; HRMS (EI) m/z calcd for C_28_H_27_NO_2_ [M]^+^ 409.2042, found 409.2046.
*1-[1-(4-Aminophenyl)-5-benzyl-2-methyl-4-(thiophen-2-yl)-1H-pyrrol-3-yl]ethan-1-one]* (**5bad**): Propargylic alcohol **1b** (33 mg, 0.15 mmol), acetylacetone (**2a**) (16 mg, 0.15 mmol), 1,3-phenylenediamine (**4d**) (34 mg, 0.31 mmol), AuBr_3_ (3.4 mg, 0.008 mmol, 5 mol%) and AgOTf (6.0 mg, 0.023 mmol, 15 mol%) in [EMIM][NTf_2_] (1 mL) furnished pyrrole **5bad** (31 mg, yield = 52%) as a colorless oil; IR (ATR) 3366, 2923, 2854, 1717, 1685, 1647, 1559, 1541, 1518, 1457, 1411 cm^−1^; ^1^H-NMR (300 MHz, CDCl_3_) δ 7.36–7.27 (5H, m), 7.08–7.02 (3H, m), 6.69 (2H, d, *J* = 8.8 Hz), 6.66 (2H, dd, *J* = 8.0, 2.8 Hz), 6.58 (2H, d, *J* = 8.8 Hz), 3.78 (2H, br s), 3.65 (2H, s), 2.24 (3H, s), 1.94 (3H, s); ^13^C-NMR (75 MHz, CDCl_3_) δ 197.3, 139.8, 136.9, 136.4, 130.6, 129.8, 129.8, 129.2, 128.3, 128.1, 127.9, 126.8, 125.7, 123.4, 121.5, 115.2, 31.0, 30.7, 13.0 (overlapped); HRMS (EI) m/z calcd for C_24_H_22_N_2_OS [M]^+^ 386.1453, found 386.1447.
*1-{1-[4-Aminophenyl]-4-[benzo(b)thiophen-3-yl]-5-benzyl-2-methyl-1H-pyrrol-3-yl}ethan-1-one* (**5cad**): Propargylic alcohol **1c** (42 mg, 0.16 mmol), acetylacetone (**2a**) (16 mg, 0.16 mmol), 1,3-phenylenediamine (**4d**) (34 mg, 0.32 mmol), AuBr_3_ (3.4 mg, 0.008 mmol, 5 mol%) and AgOTf (6.1 mg, 0.024 mmol, 15 mol%) in [EMIM][NTf_2_] (1 mL) furnished pyrrole **5cad** (40 mg, yield = 65%) as a colorless oil; IR (ATR) 3363, 2923, 2854, 1710, 1629, 1515, 1455, 1411 cm^−1^; ^1^H NMR (400 MHz, CDCl_3_) δ 7.90–7.86 (1H, m), 7.68–7.64 (1H, m), 7.38–7.34 (2H, m), 7.32 (1H, s), 7.03–6.99 (3H, m), 6.76 (2H, dd, *J* = 8.4, 2.4 Hz) 6.65–6.60 (3H, m), 6.57 (1H, ddd, *J* = 8.2, 8.1, 2.6 Hz), 3.83 (2H, br s), 3.67 (1H, d, *J* = 16.0 Hz), 3.55 (1H, d, *J* = 16.0 Hz), 2.31 (3H, s), 1.83 (3H, s); ^13^C NMR (100 MHz, CDCl_3_) δ 196.9, 146.6, 140.3, 139.8, 139.5, 137.2, 132.3, 131.4, 129.2, 129.1, 128.1, 127.9, 127.6, 125.7, 124.6, 124.4, 123.3, 122.7, 121.6, 115.7, 115.0, 31.1, 29.8, 13.2; HRMS (EI) m/z calcd for C_28_H_24_N_2_OS [M]^+^ 436.1609, found 436.1603.

### 3.3. Procedure of Gold(III)-Catalyzed Reaction from Hydrated Propargylic Substitution Product ***3″aa*** and Aniline ***4a***

5 mol% AuBr_3_ and 15 mol% AgOTf were added at room temperature to a solution of hydrated propargylic substitution product **3″aa** and aniline **4a** in [ethylmethylimidazolium (EMIM)][NTf_2_] (1 mL). The reaction mixture was stirred at 60 °C for 3 days. After addition of diethyl ether (5 mL × 5), the organic layer was extracted and the combined organic layers were washed with brine and dried over Na_2_SO_4_. The solvent was removed in vacuo and the crude product was subjected to SiO_2_ column chromatography (hexane/AcOEt = 20:1) to give pyrrole **5aaa** in 70% yield.

## 4. Conclusions

We present an efficient synthesis of polysubstituted pyrroles in ionic liquids via a sequential reaction of propargyl substitution/hydration/amination/cycloisomerization using a gold catalyst. This reaction provides an environmentally friendly alternative approach for synthesizing various types of polysubstituted pyrroles. We are currently applying this method to synthesize biologically important compounds possessing a pyrrole skeleton. Further experimental and theoretical studies on the reaction mechanism are also underway.

## Figures and Tables

**Table 1:** Optimization of the reaction conditions for the gold-catalyzed propargylic substitution/hydration/amination/cycloisomerization sequence for the synthesis of polysubstituted pyrrole **5aaa**.

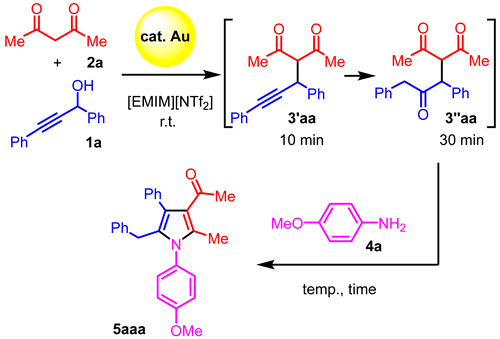				
**Entry**	**Cat. Au**	**Temp.**	**Time**	**5aaa Yield**
1	AuCl (5)	100 °C	1 day	Trace
2	AuBr_3_ (5)	100 °C	1 day	No reaction
3	AuCl (5)/AgOTf (5)	100 °C	1 day	53%
4	AuBr_3_ (5)/AgOTf (15)	100 °C	1 day	72%
5	AuBr_3_ (5)/AgOTf (15)	60 °C	3 days	93%
6	AuBr_3_ (2.5)/AgOTf (7.5)	100 °C	1 day	26%
7	AgOTf (15 mol%)	60 °C	3 days	trace

**Table 2:** Solvent effect in gold-catalyzed reaction for the synthesis of polysubstituted pyrrole **5aaa**.

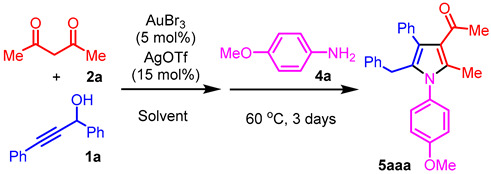		
**Entry**	**Solvent**	**5aaa Yield**
1	[EMIM][NTf_2_]	93%
2	[EMIM][OTf]	n.d.
3	[EMIM][HSO_4_]	n.d.
4	[EMIM][MeSO_4_]	n.d.
5	[EMIM][Me_2_PO_4_]	No reaction
6	[EMIM][CH_3_CO_2_]	No reaction
7	toluene	n.d.
8	ClCH_2_CH_2_Cl	28%
9	THF	No reaction

**Table 3:** Effect of cation part of ionic liquid in gold-catalyzed reaction for the synthesis of polysubstituted pyrrole **5aaa**.

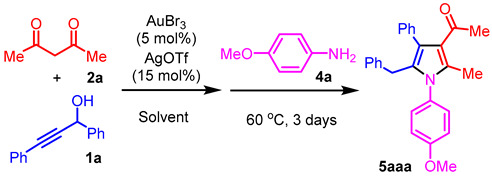		
**Entry**	**Solvent**	**5aaa Yield**
1	[EMIM][NTf_2_]	93%
2	[Butyltrimethylammonium][NTf_2_]	80%
3	[1-Butylpyridium][NTf_2_]	84%
4	[1-Butyl-1-methylpyrrolidinium][NTf_2_]	80%
5	[Tributylmethylphosphonium][NTf_2_]	86%
6	[1-Butyl-1-methylpiperidinium][NTf_2_]	67%
7	[Triethylsulfonium][NTf_2_]	n.d.
8	[1-(3-Cyanopropyl)-3-methyl-1*H*-imidazol-3-ium] [NTf_2_]	n.d.

## Data Availability

Data are contained within the article and Supplementary Materials.

## Supplementary Materials

The following supporting information can be downloaded at: https://www.mdpi.com/article/10.3390/molecules31071203/s1, ^1^H, ^13^C-NMR spectrum.
