# Supplementary figures and images for: Environmentally Friendly Synthesis of Polysubstituted Pyrroles in Ionic Liquid via Gold-Catalyzed Propargylic Substitution/Hydration/Amination/Cycloisomerization Sequence
Source: Molecules. 2026 Apr 5;31(7):1203. doi: 10.3390/molecules31071203 (PMC13074243; doi:10.3390/molecules31071203)

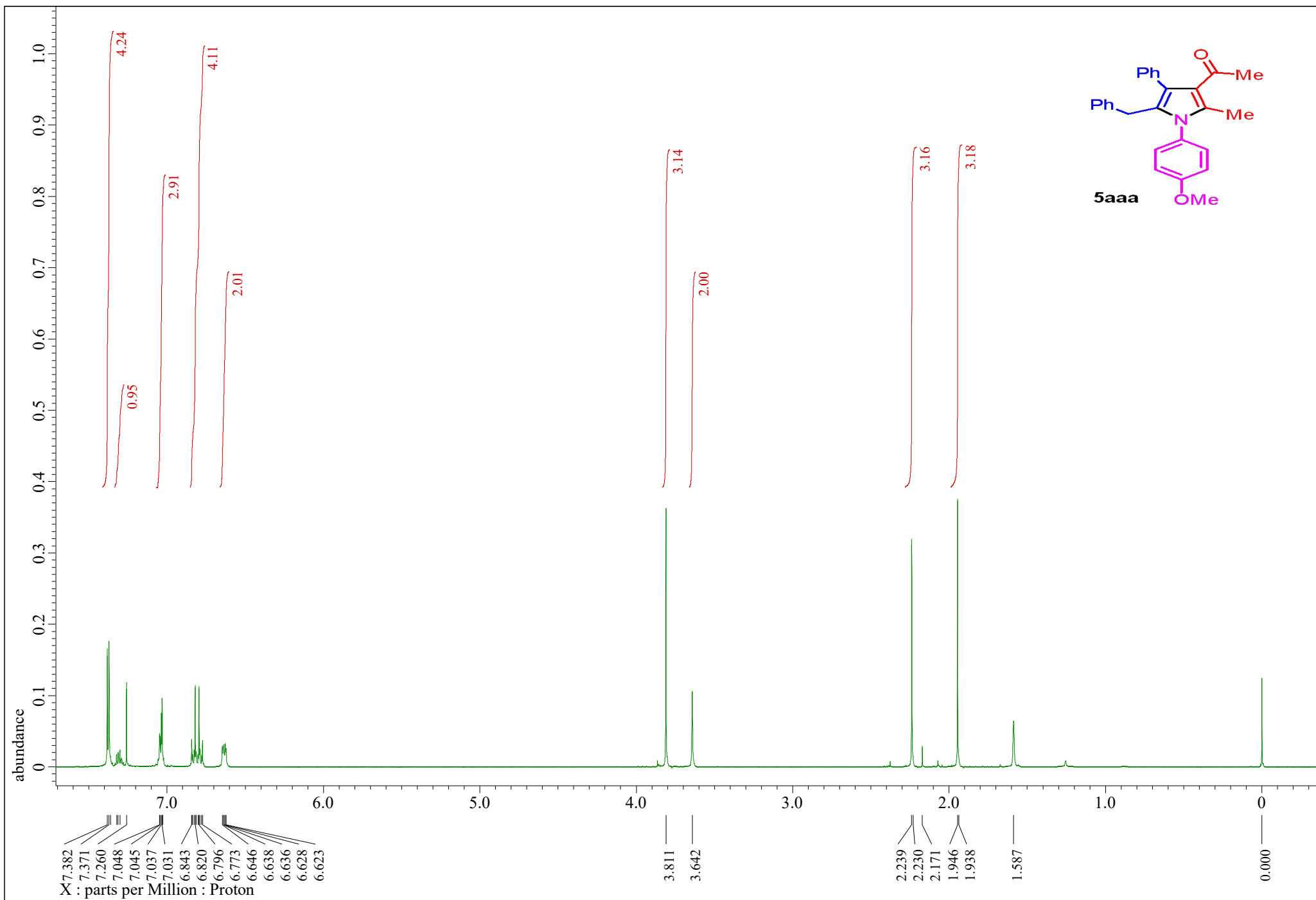

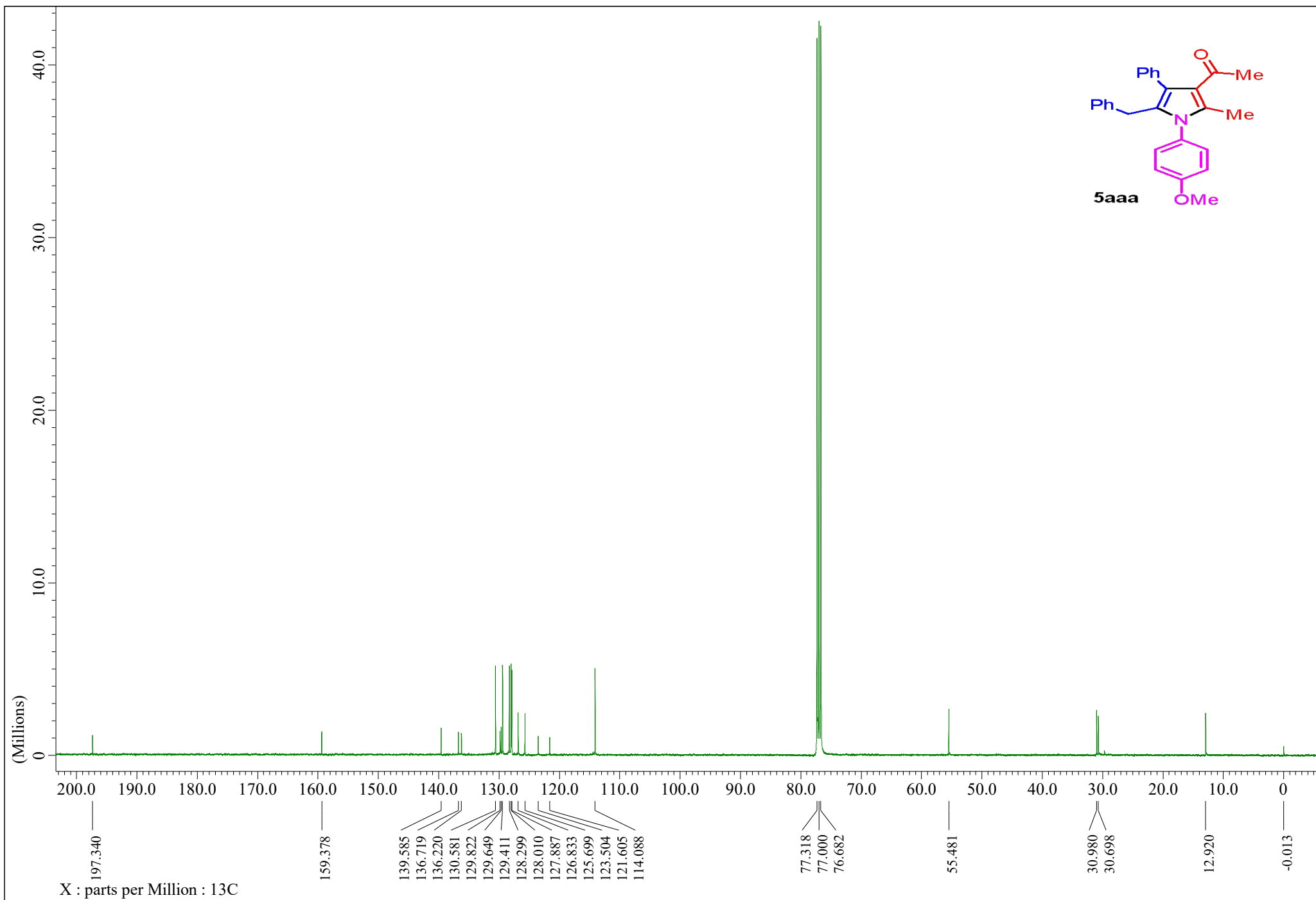

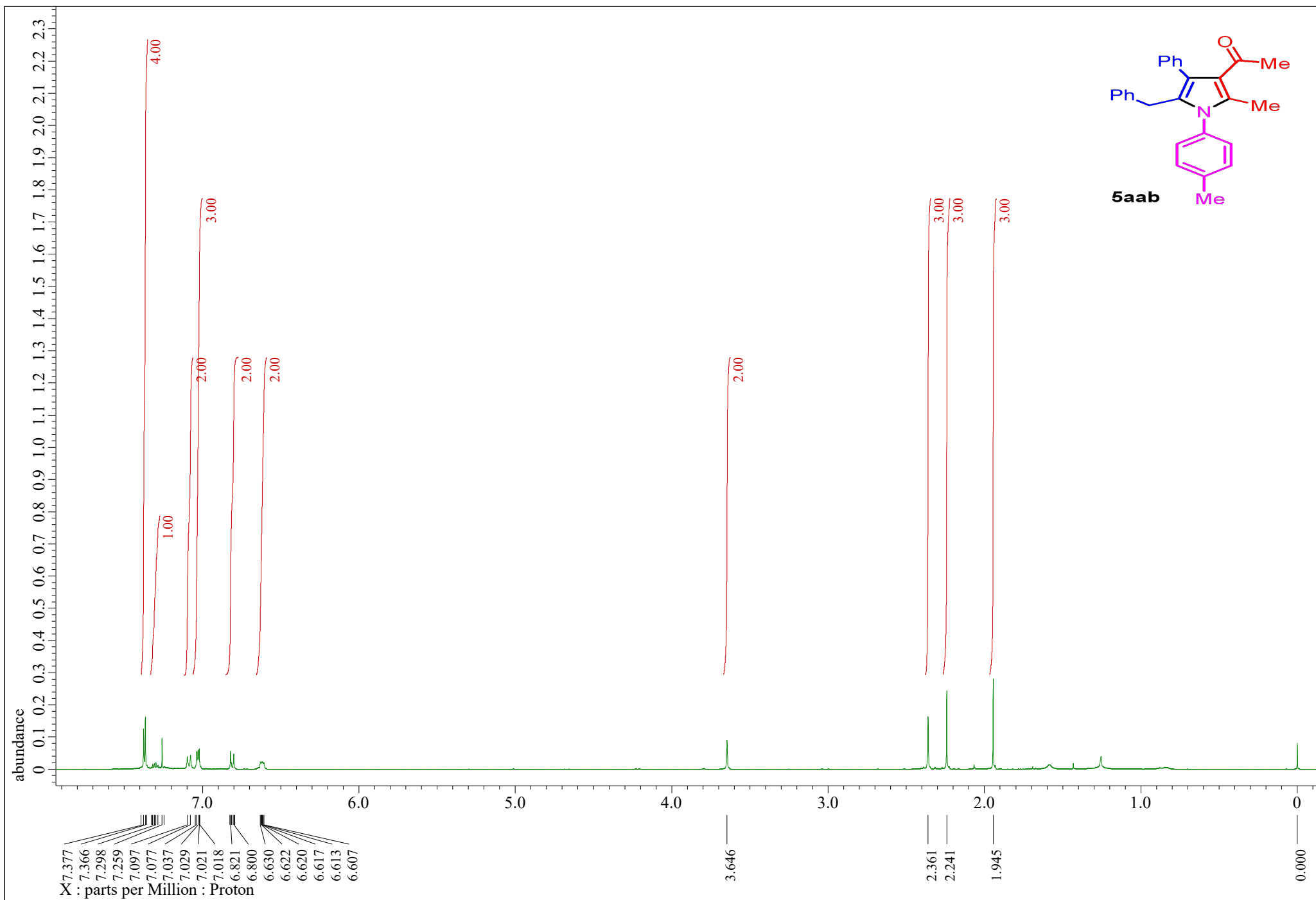

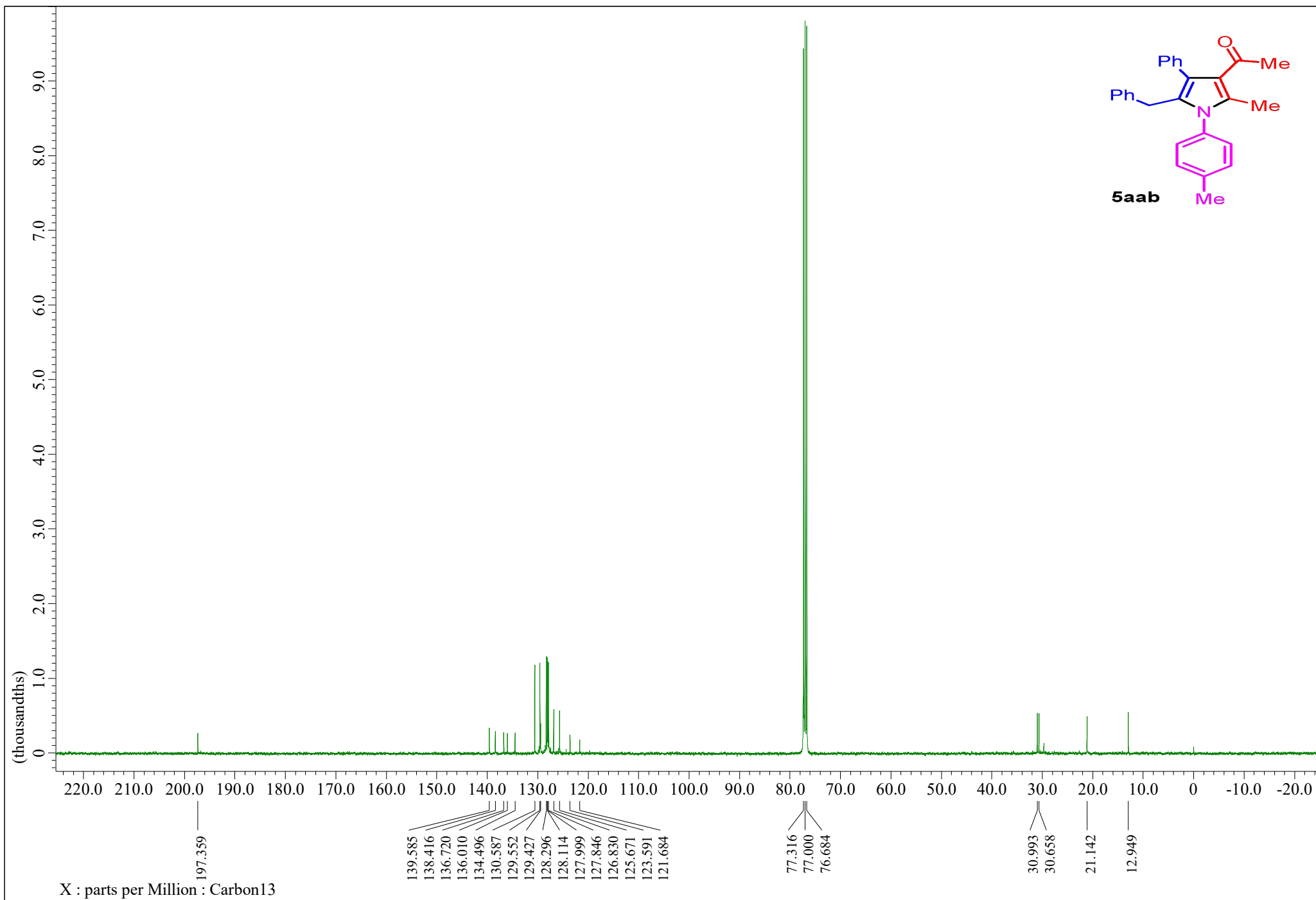

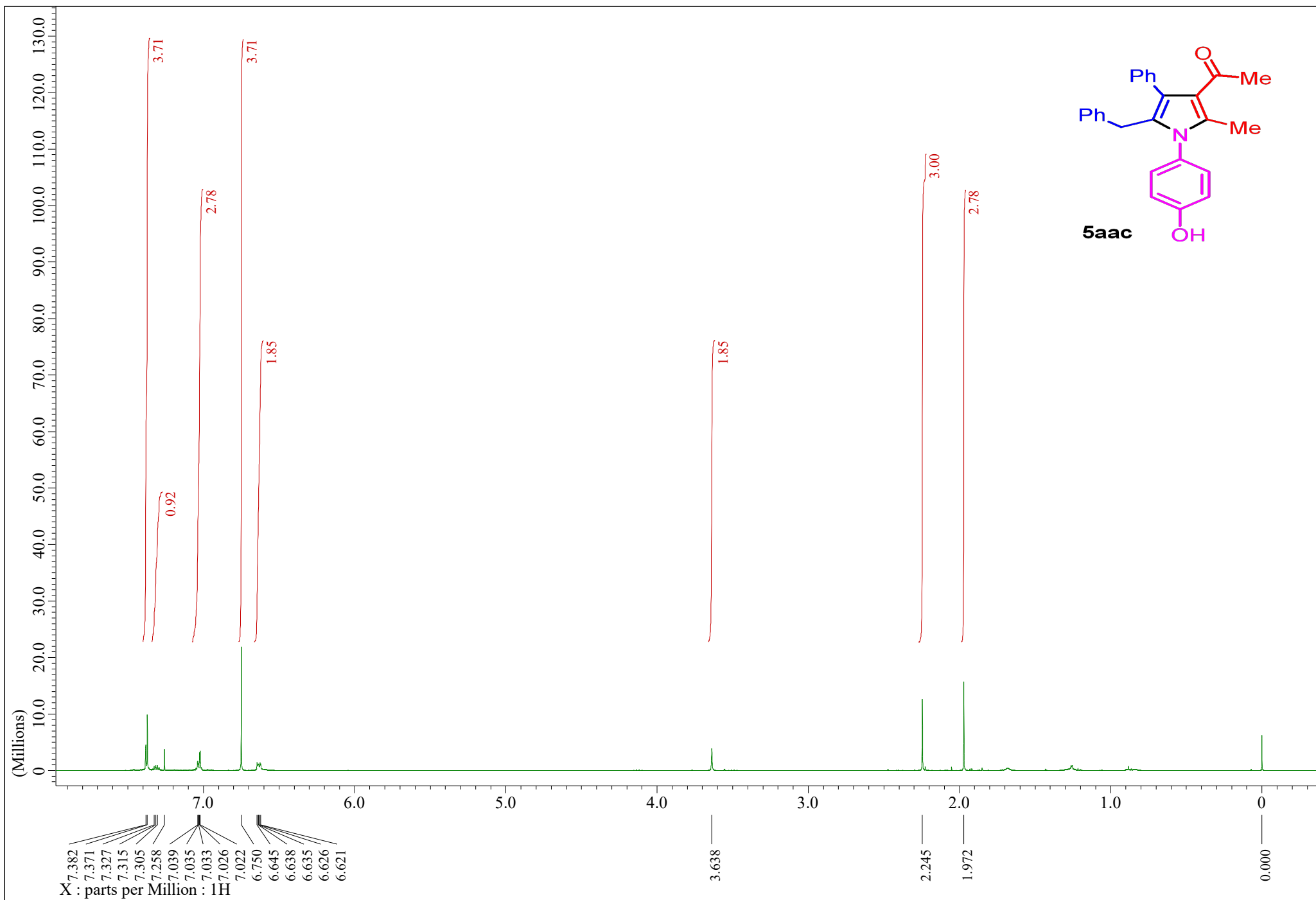

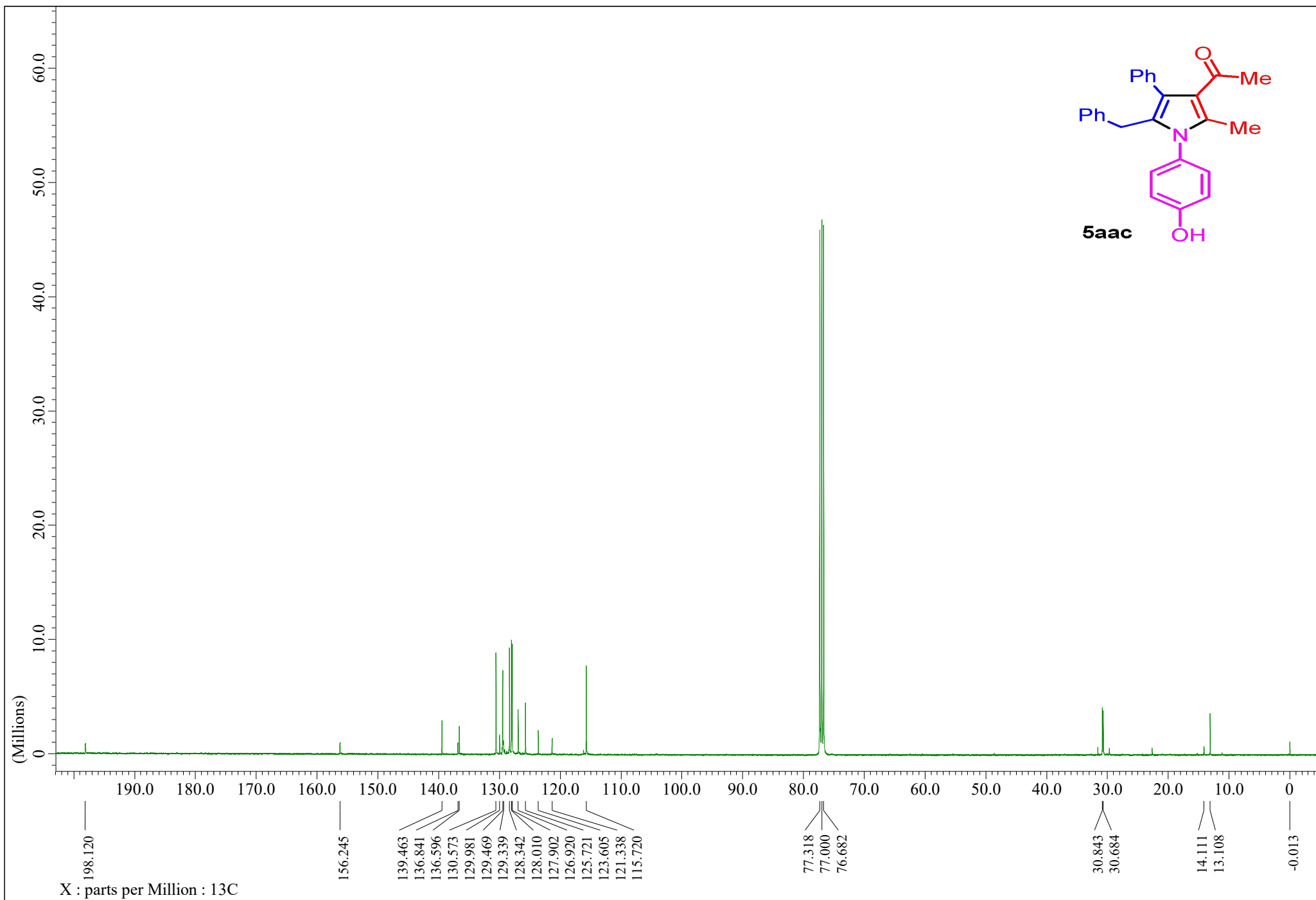

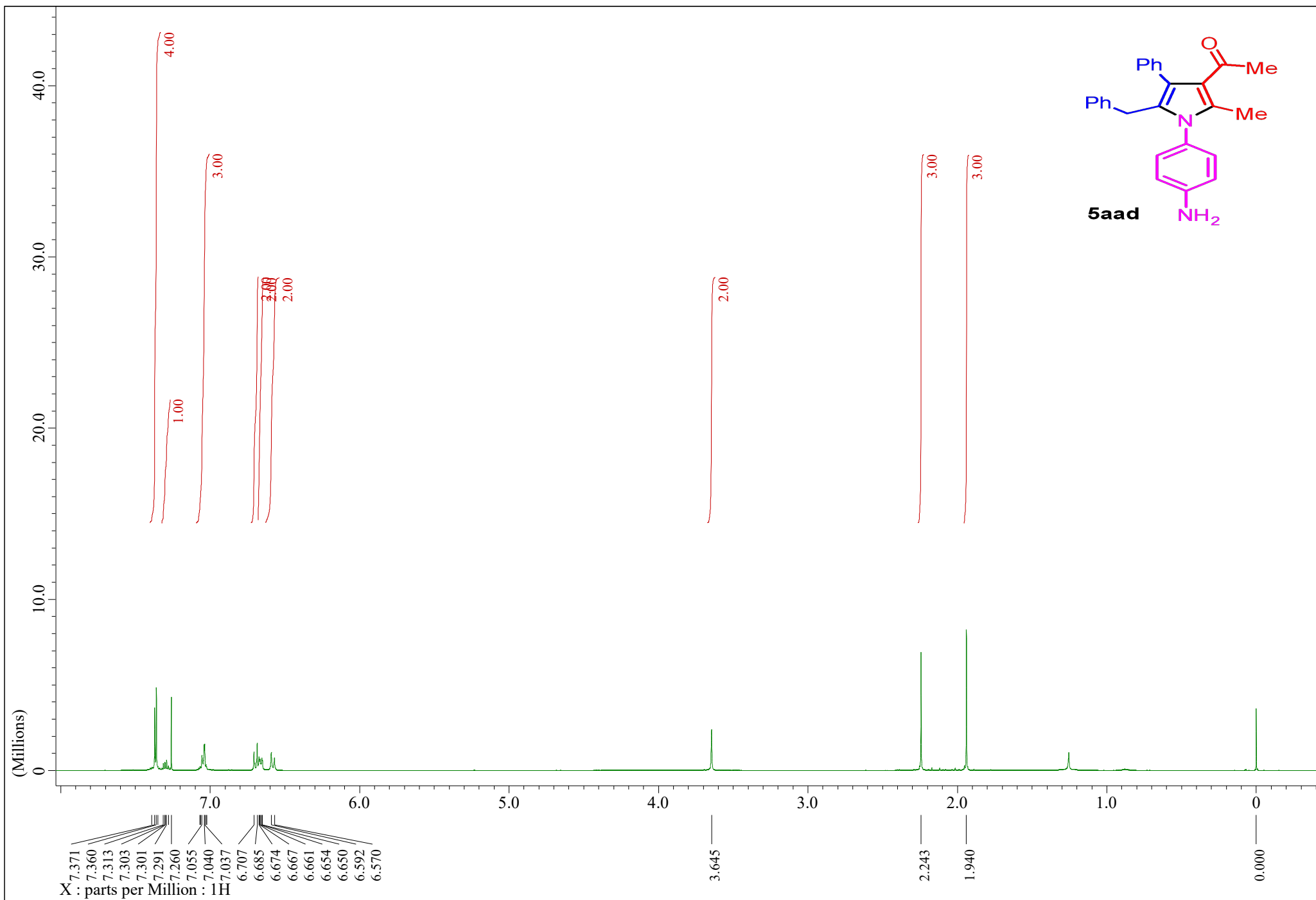

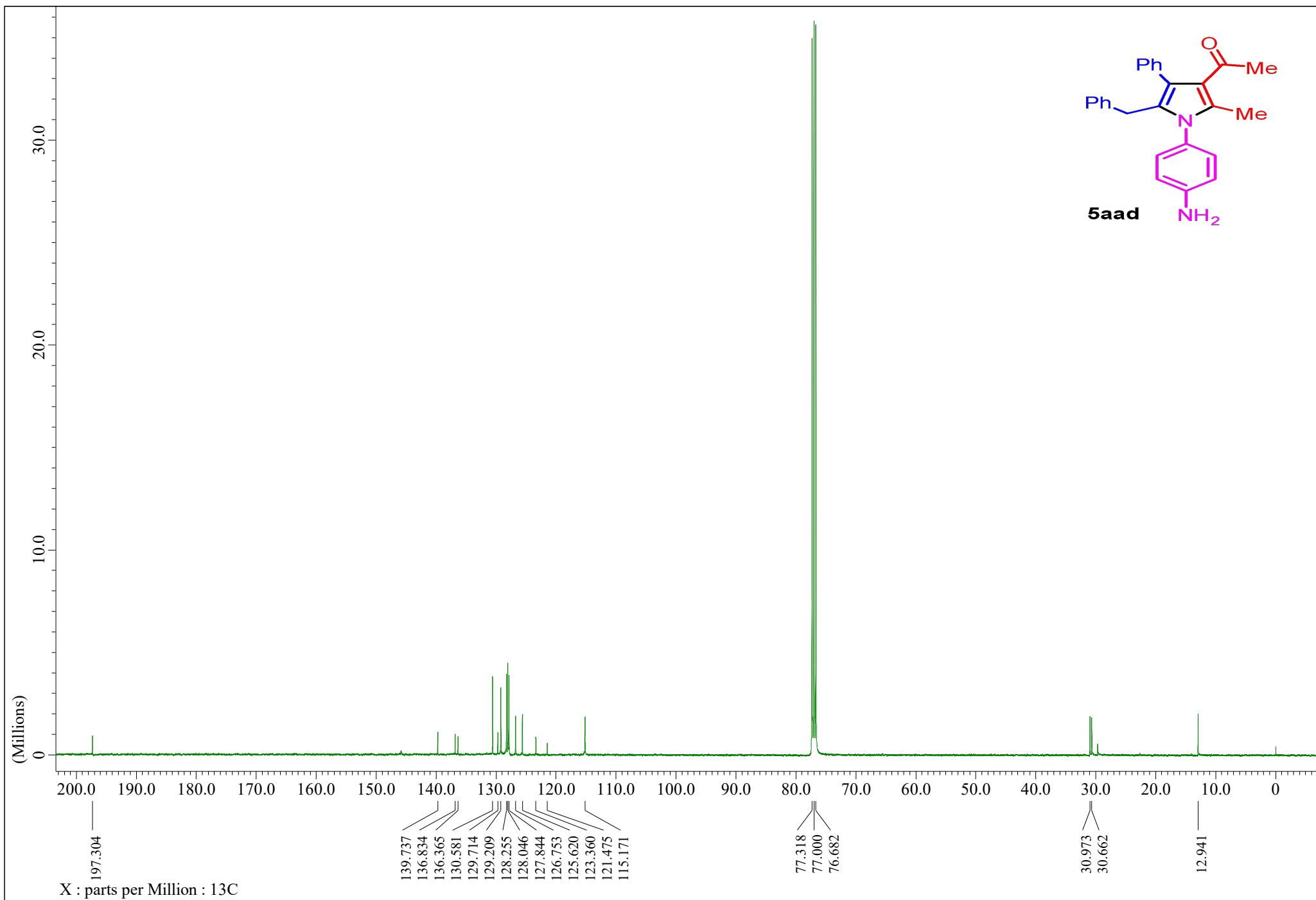

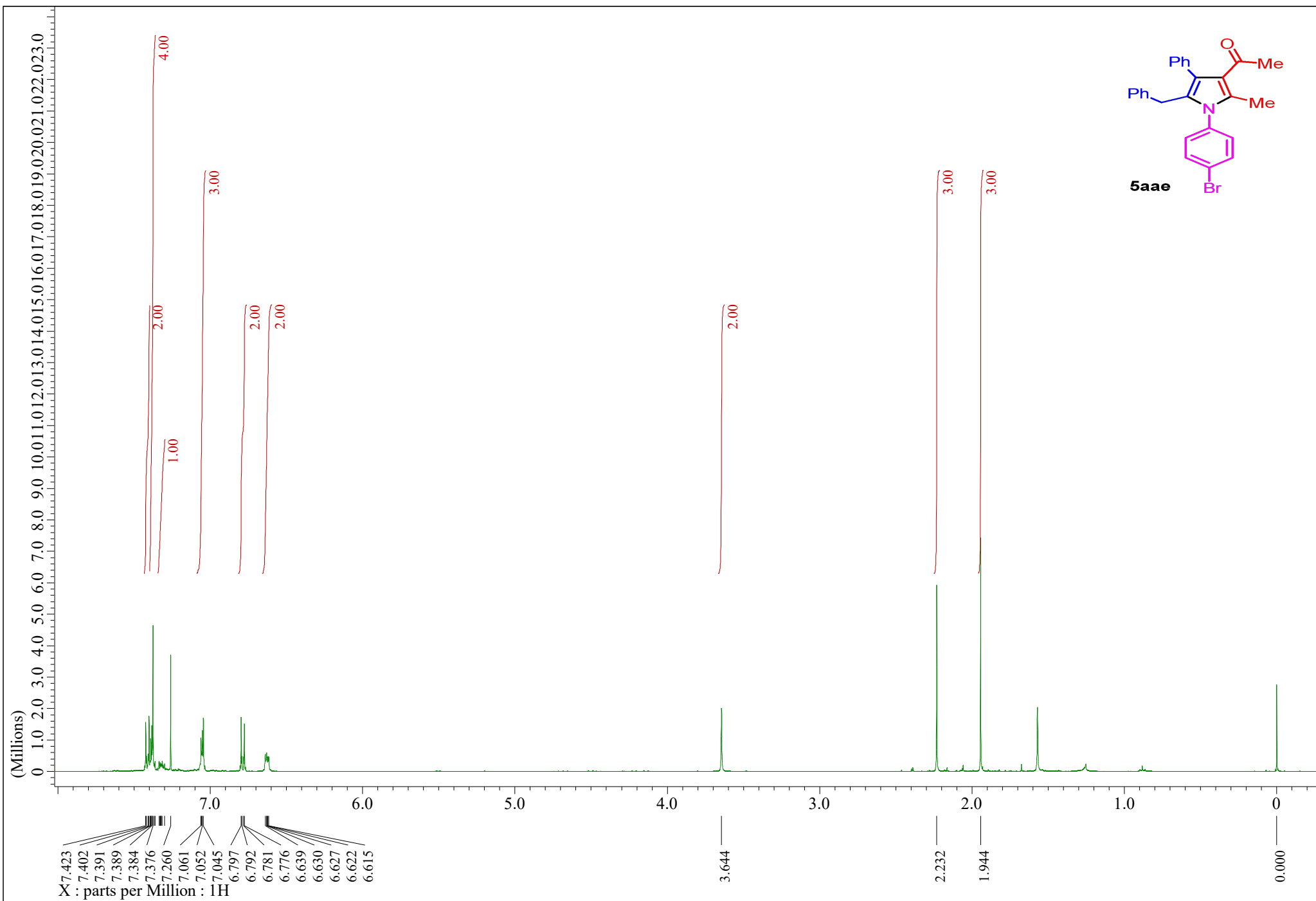

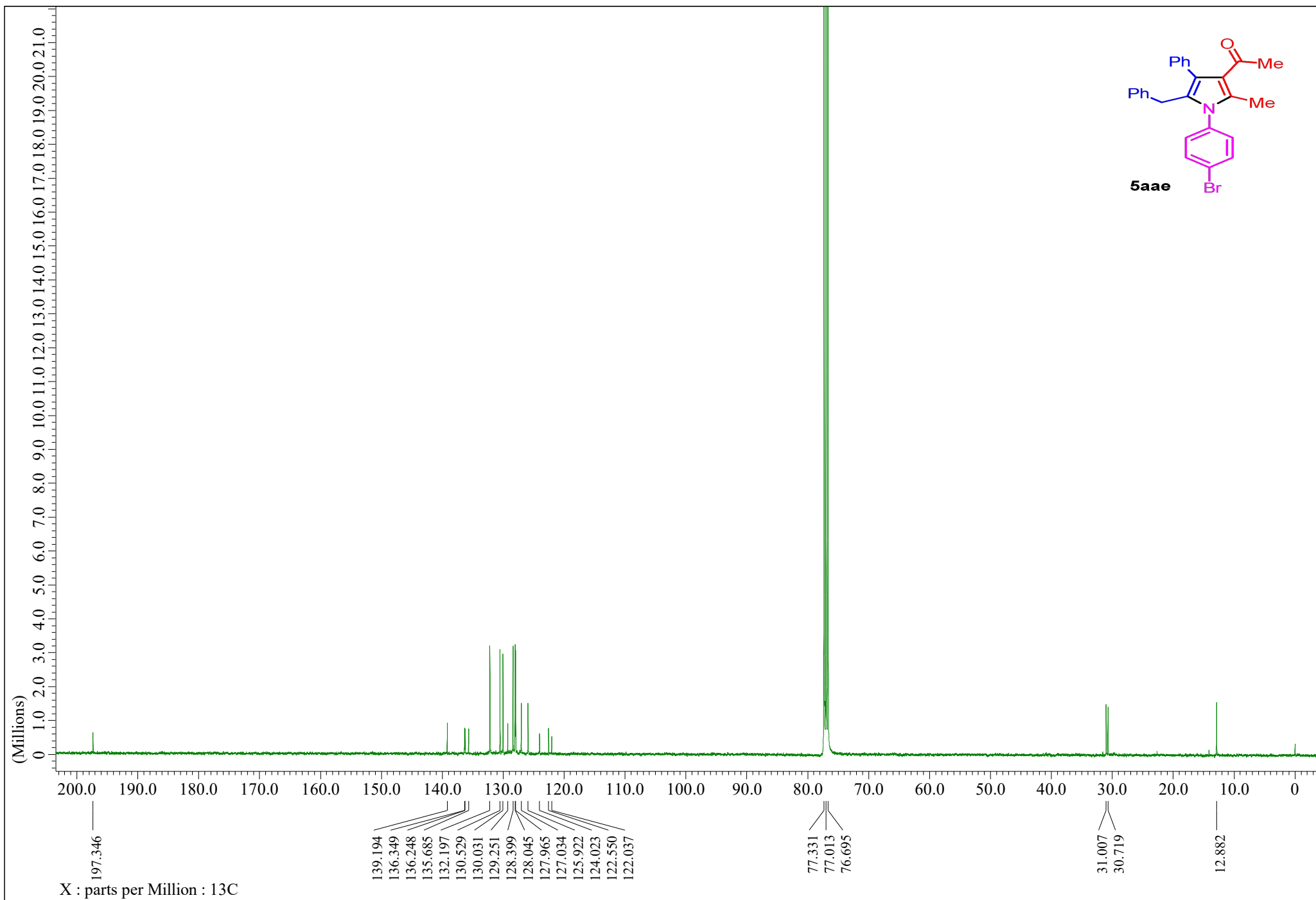

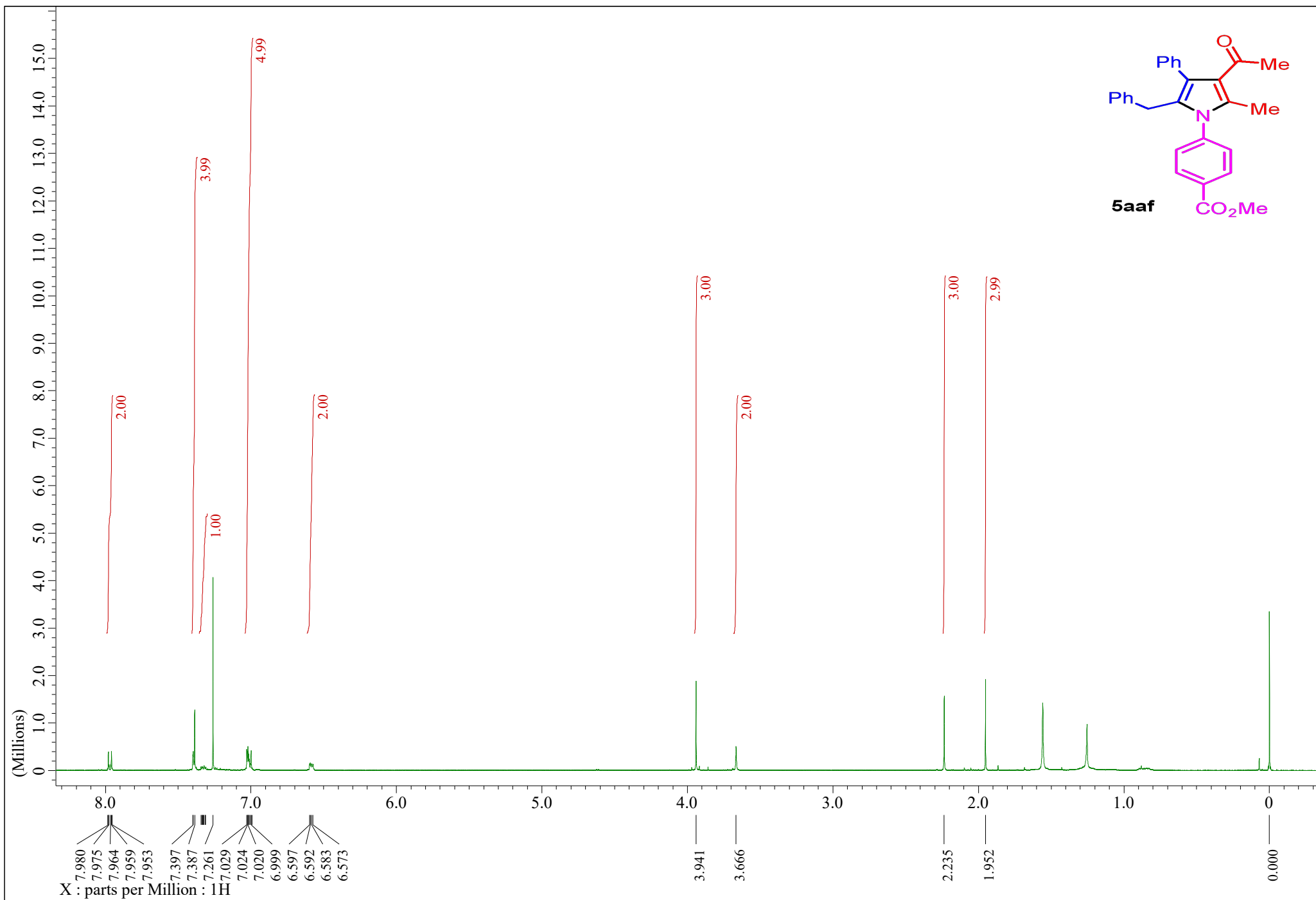

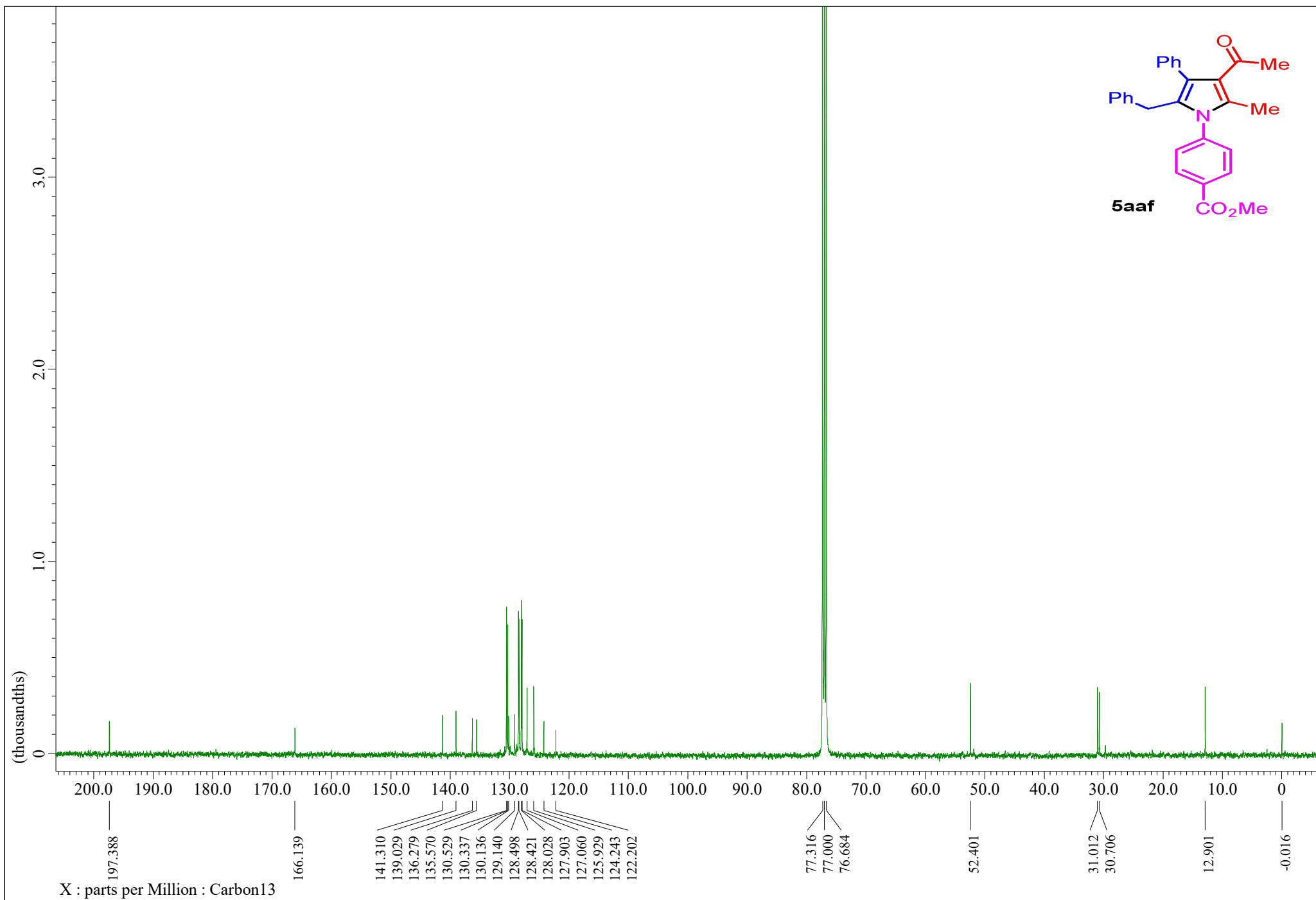

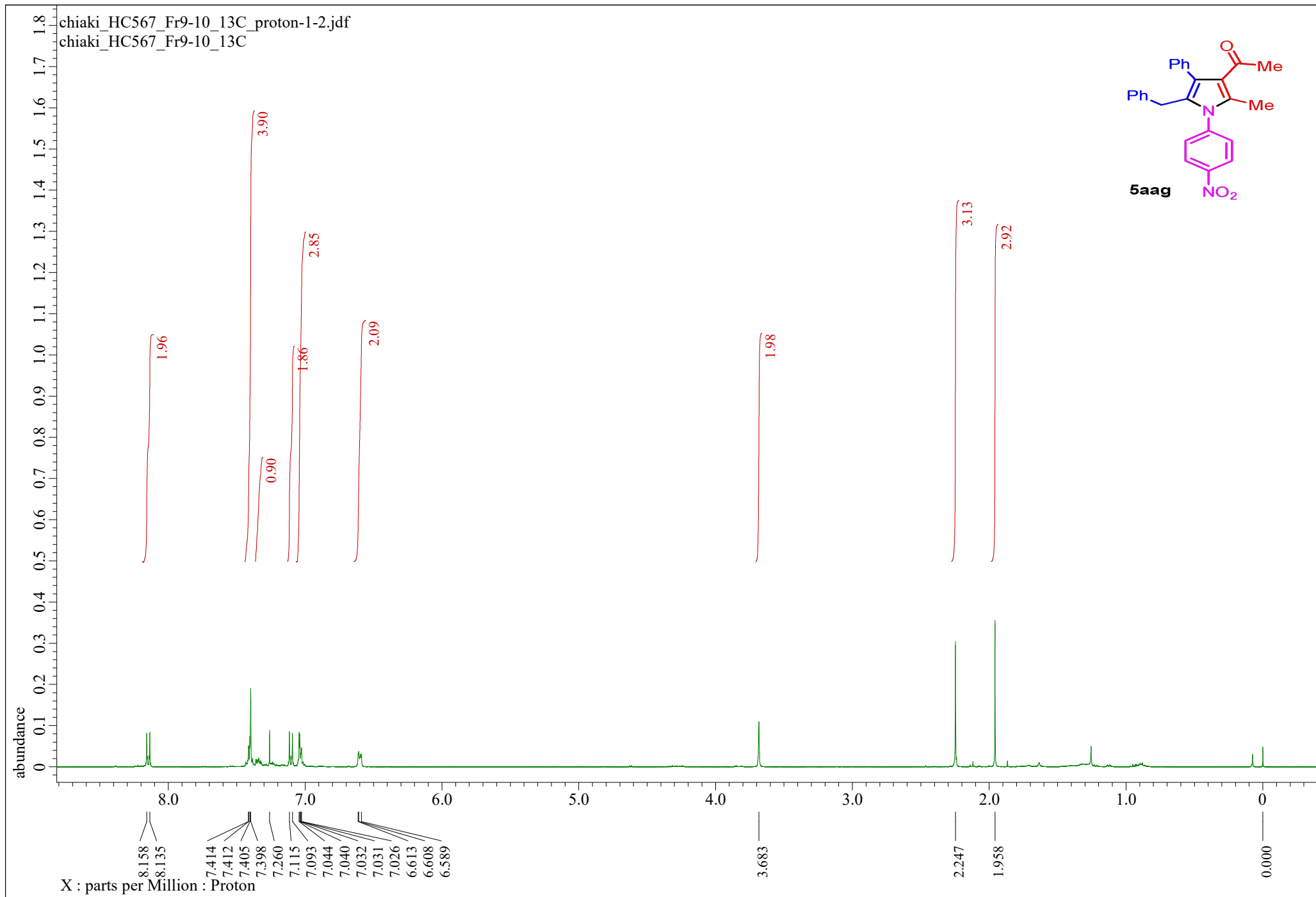

chiaki\_HC567\_Fr9-10\_13C\_carbon-1-2.jdf  
chiaki\_HC567\_Fr9-10\_13C

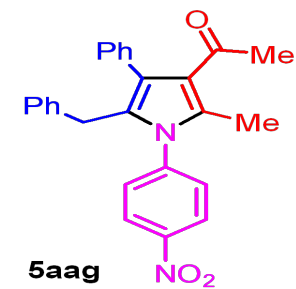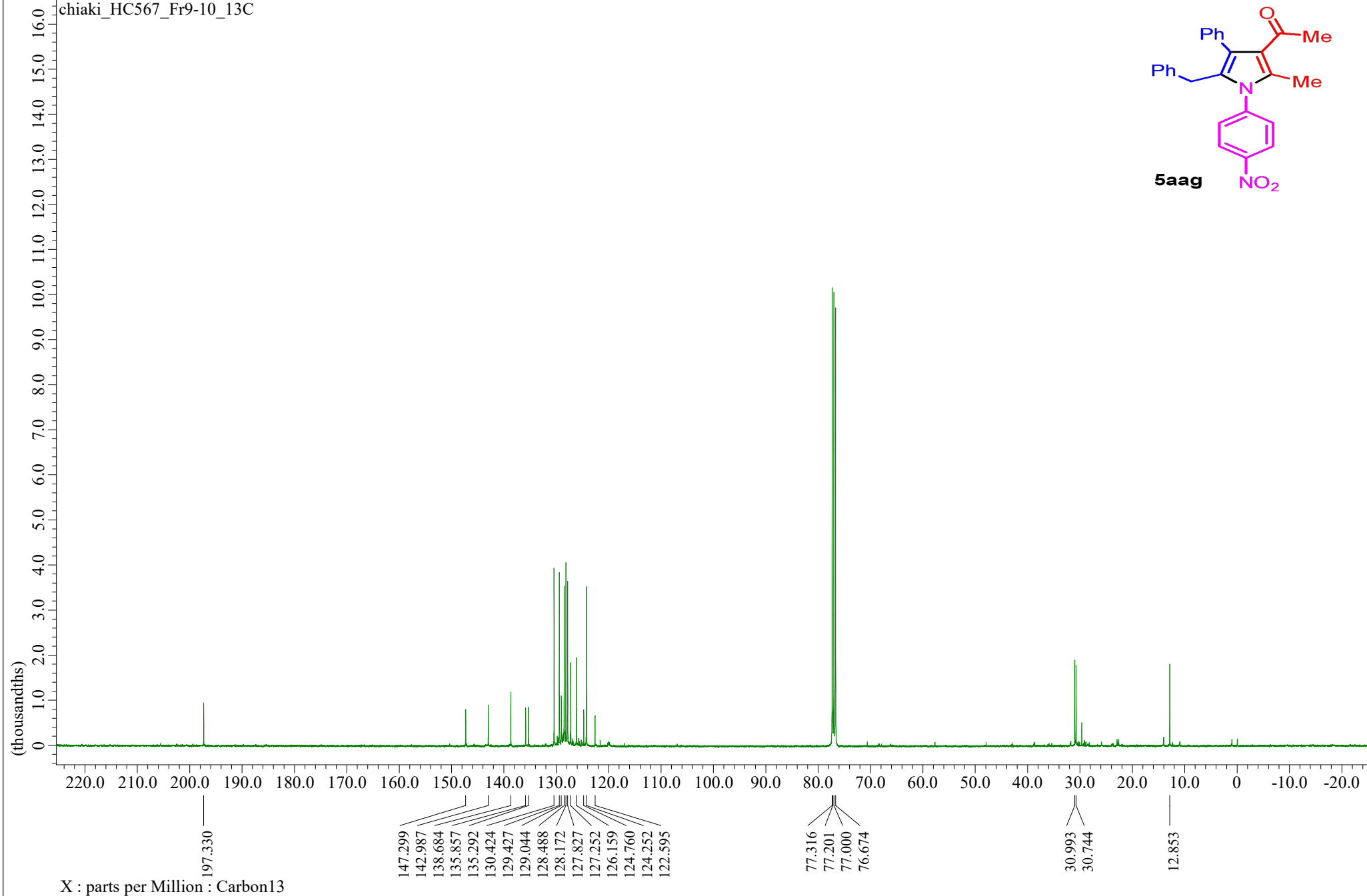

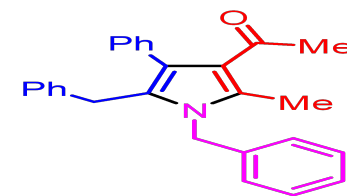

**5aam**

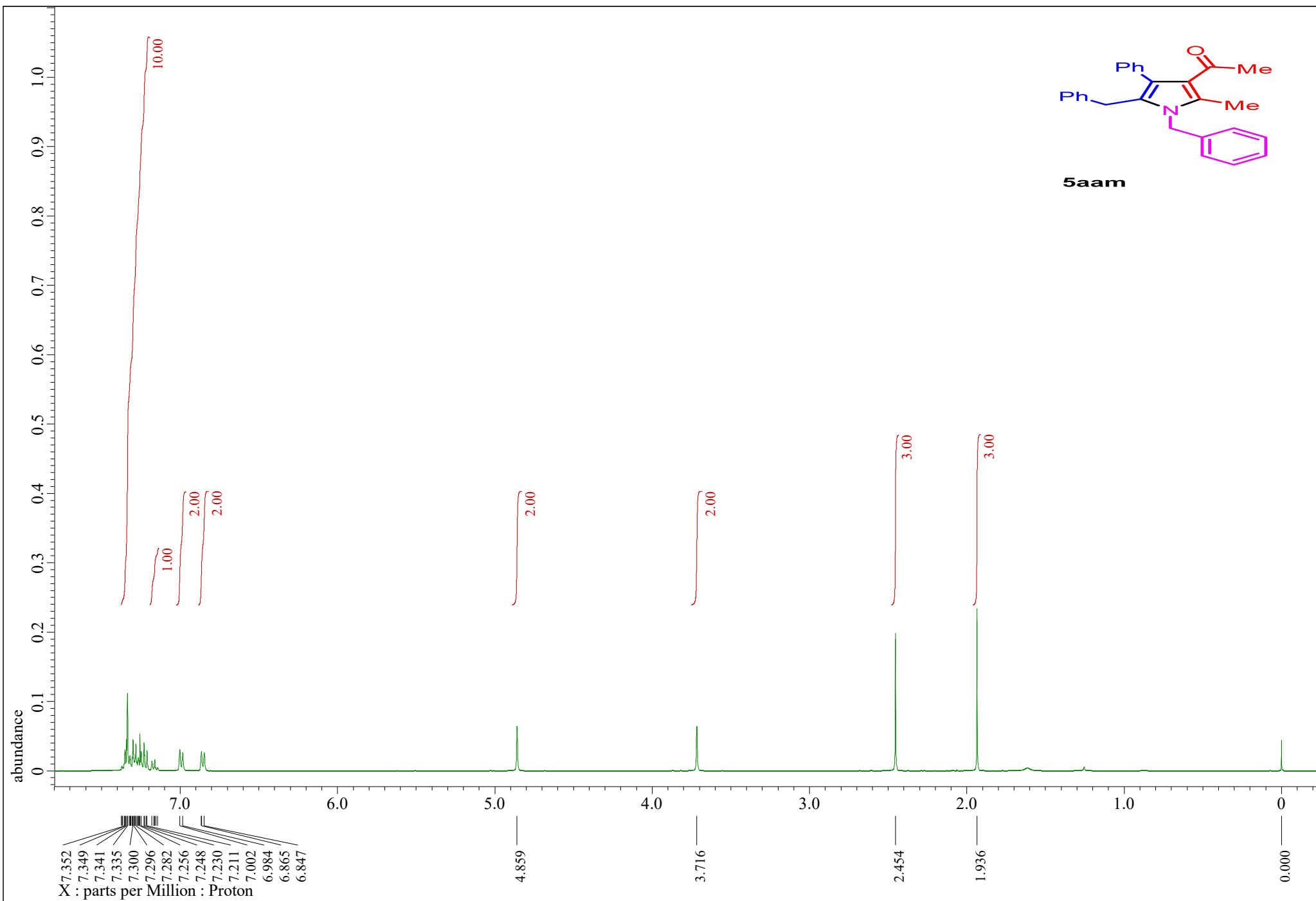

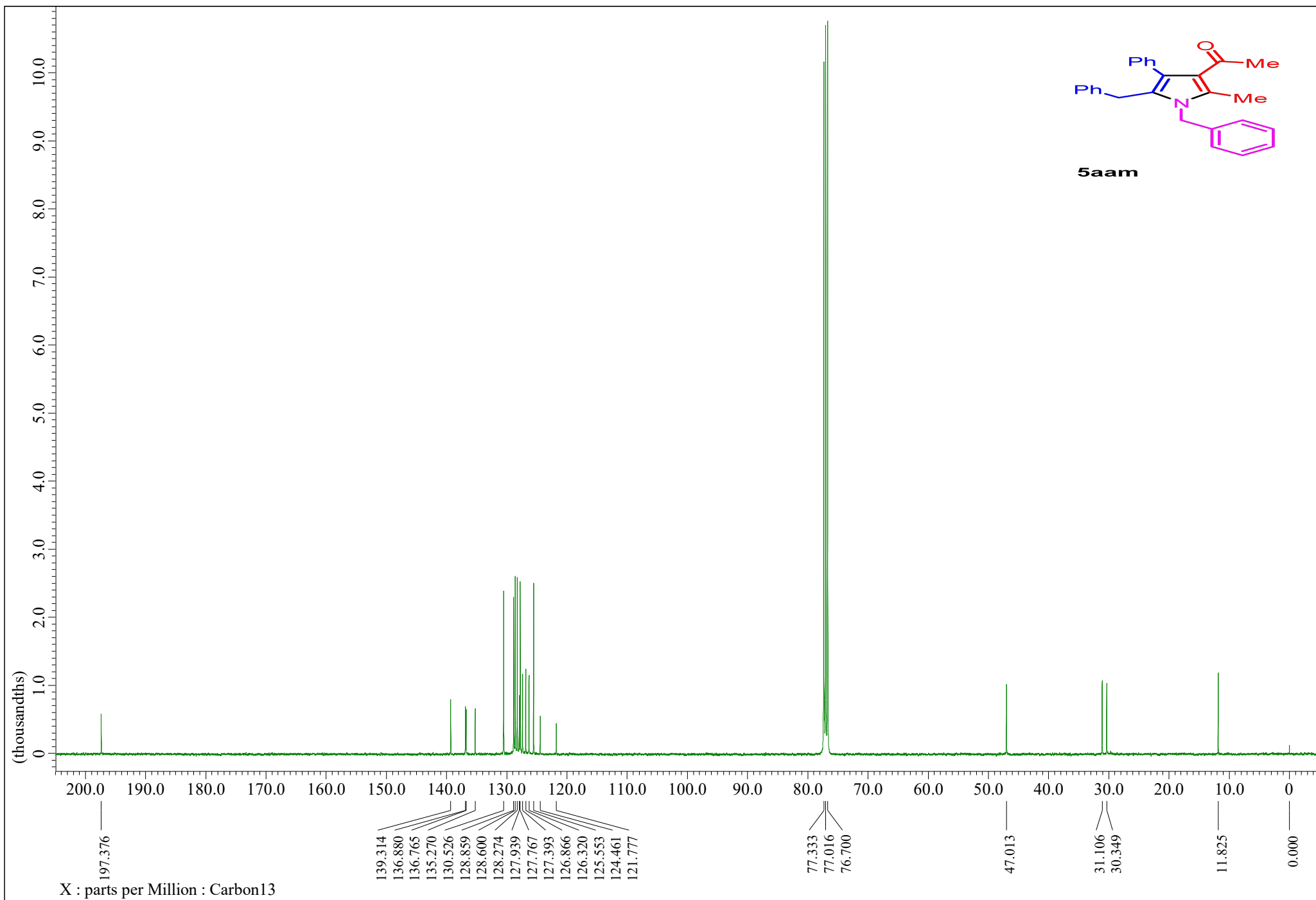

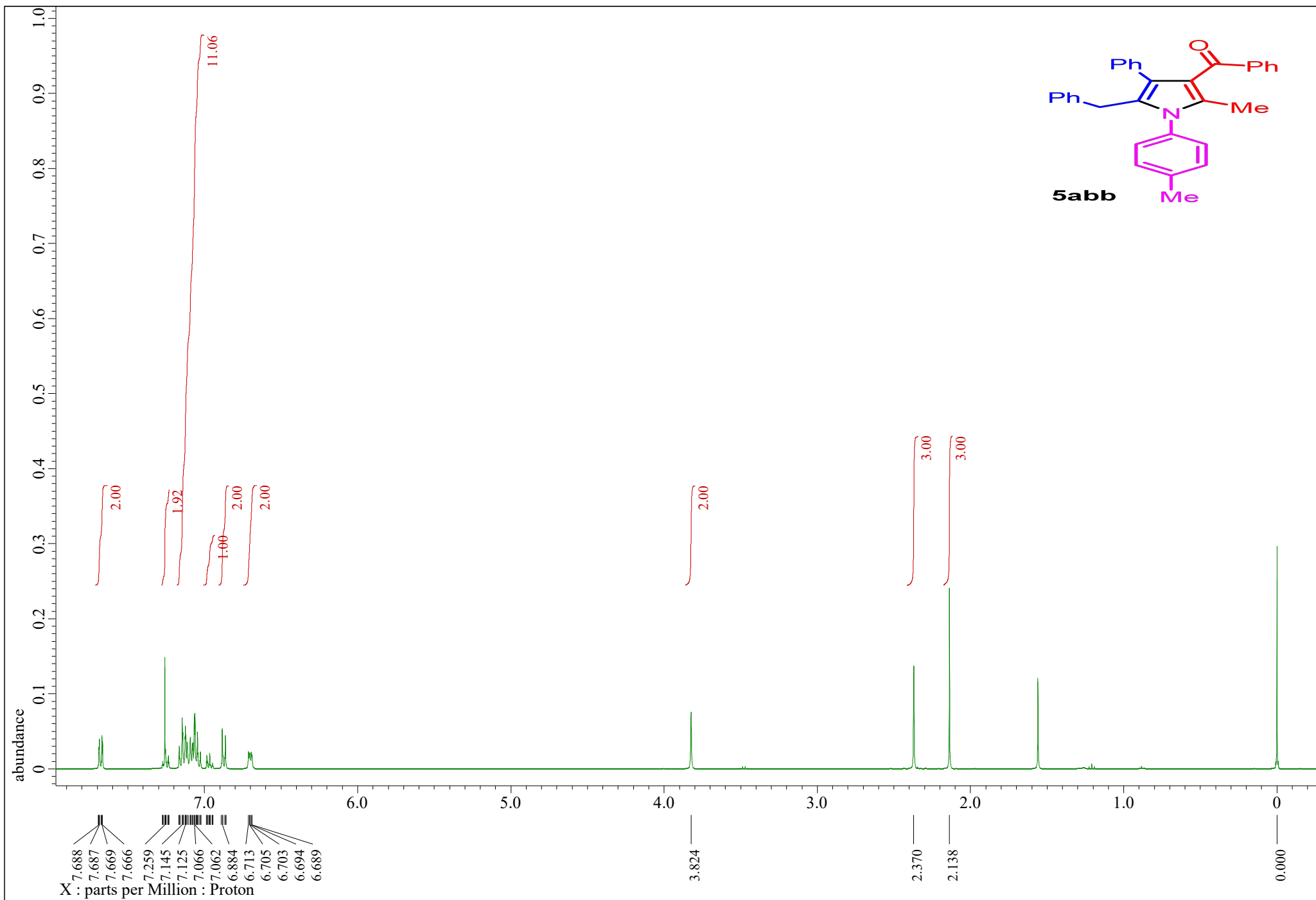

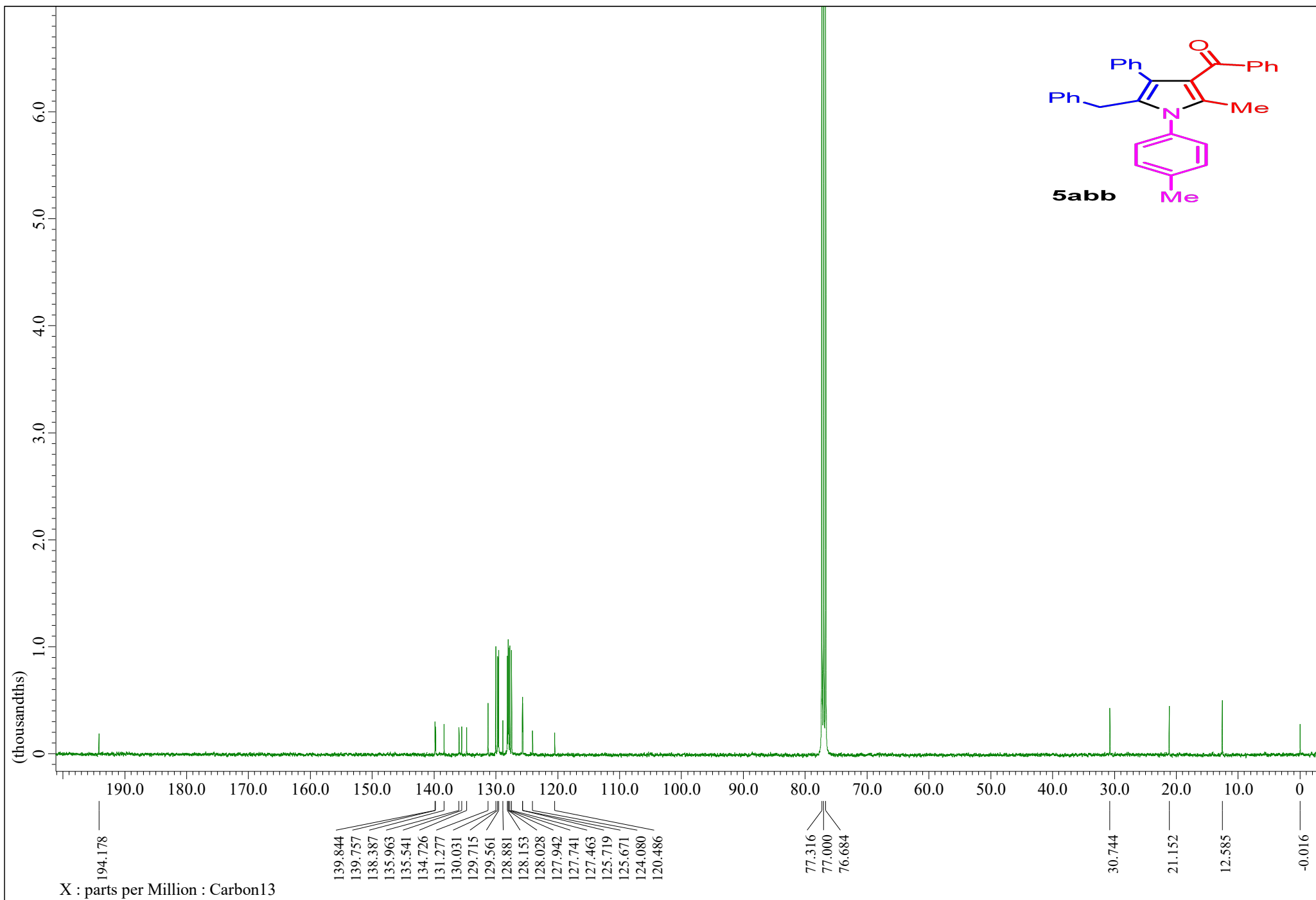

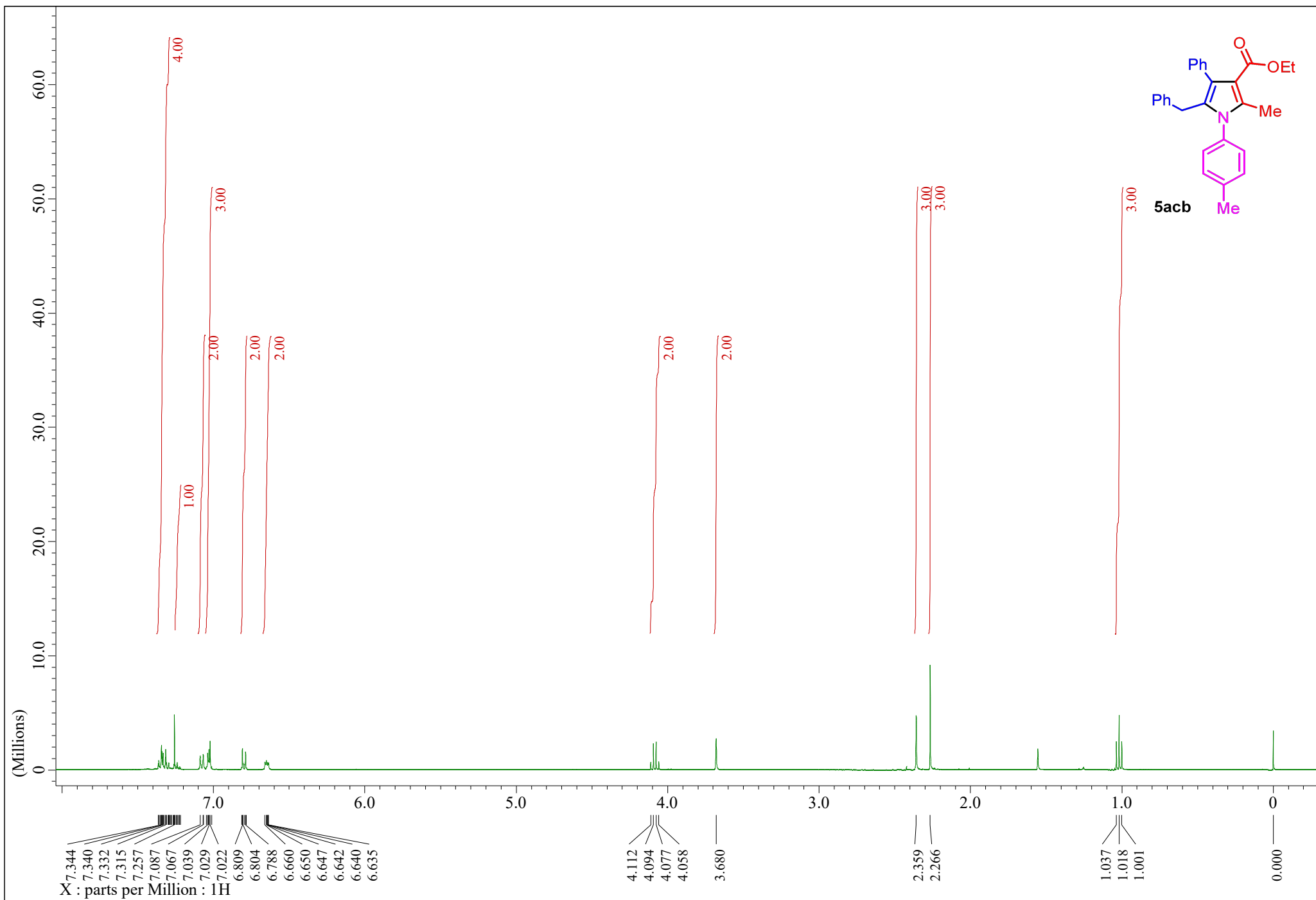

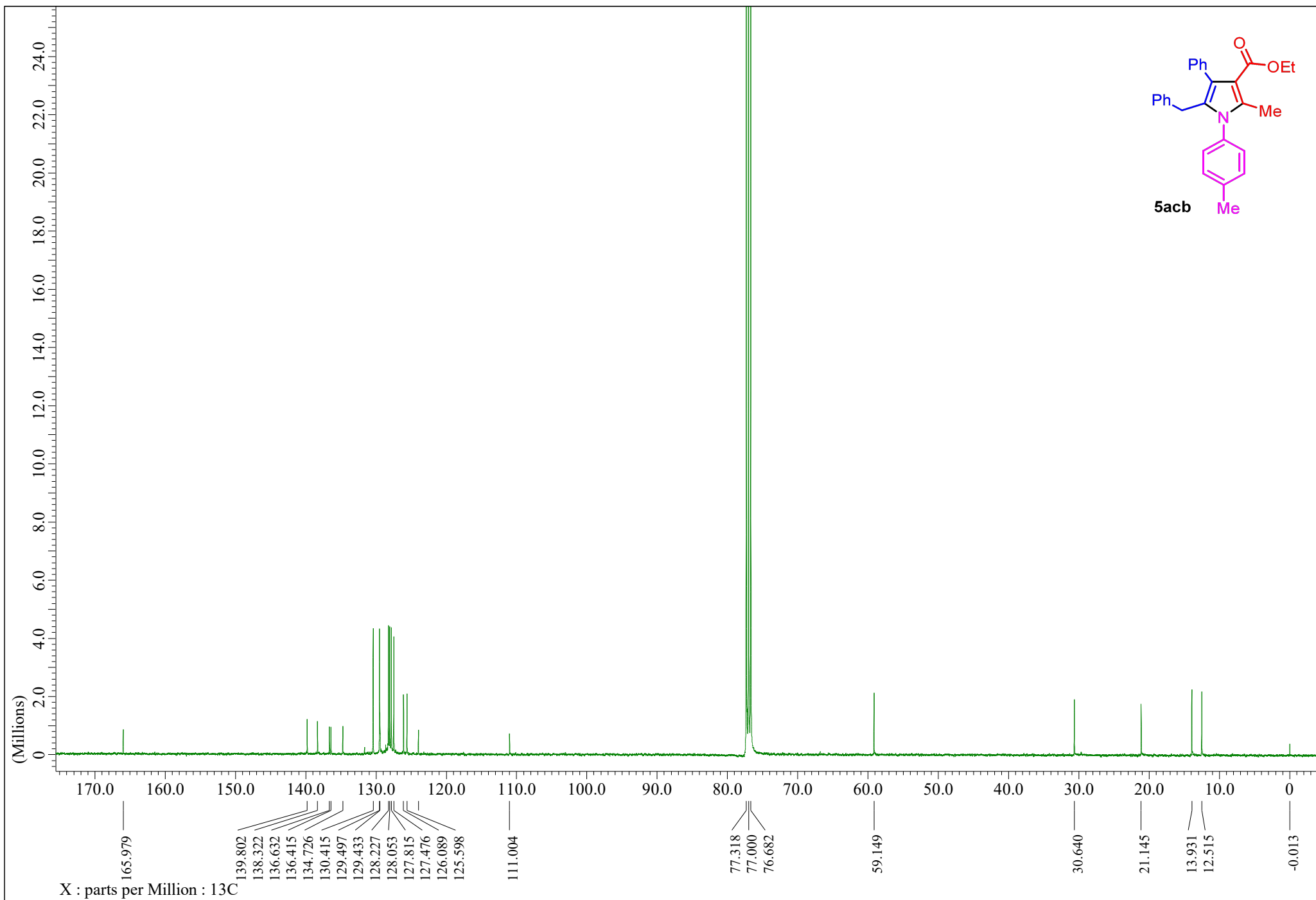

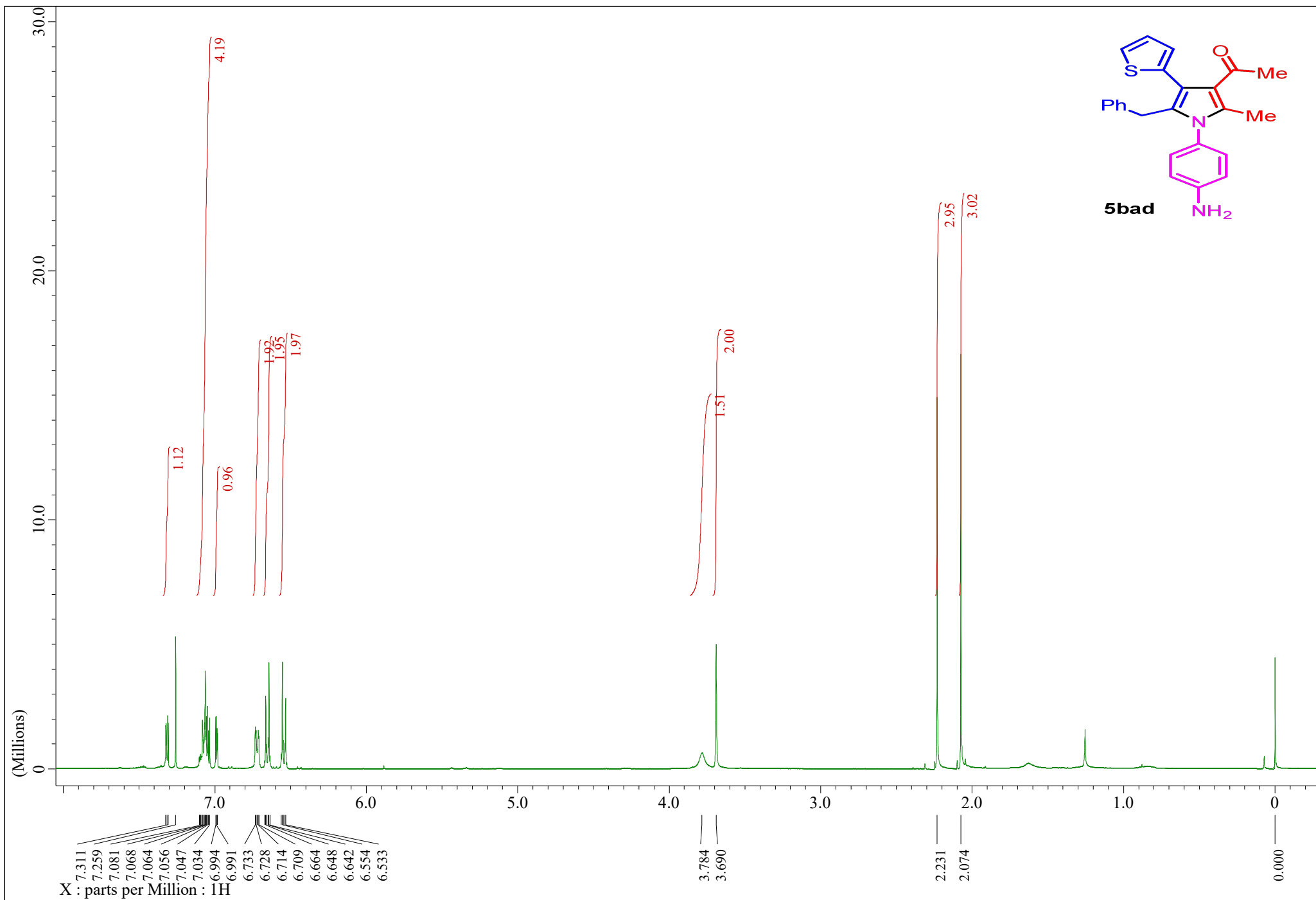

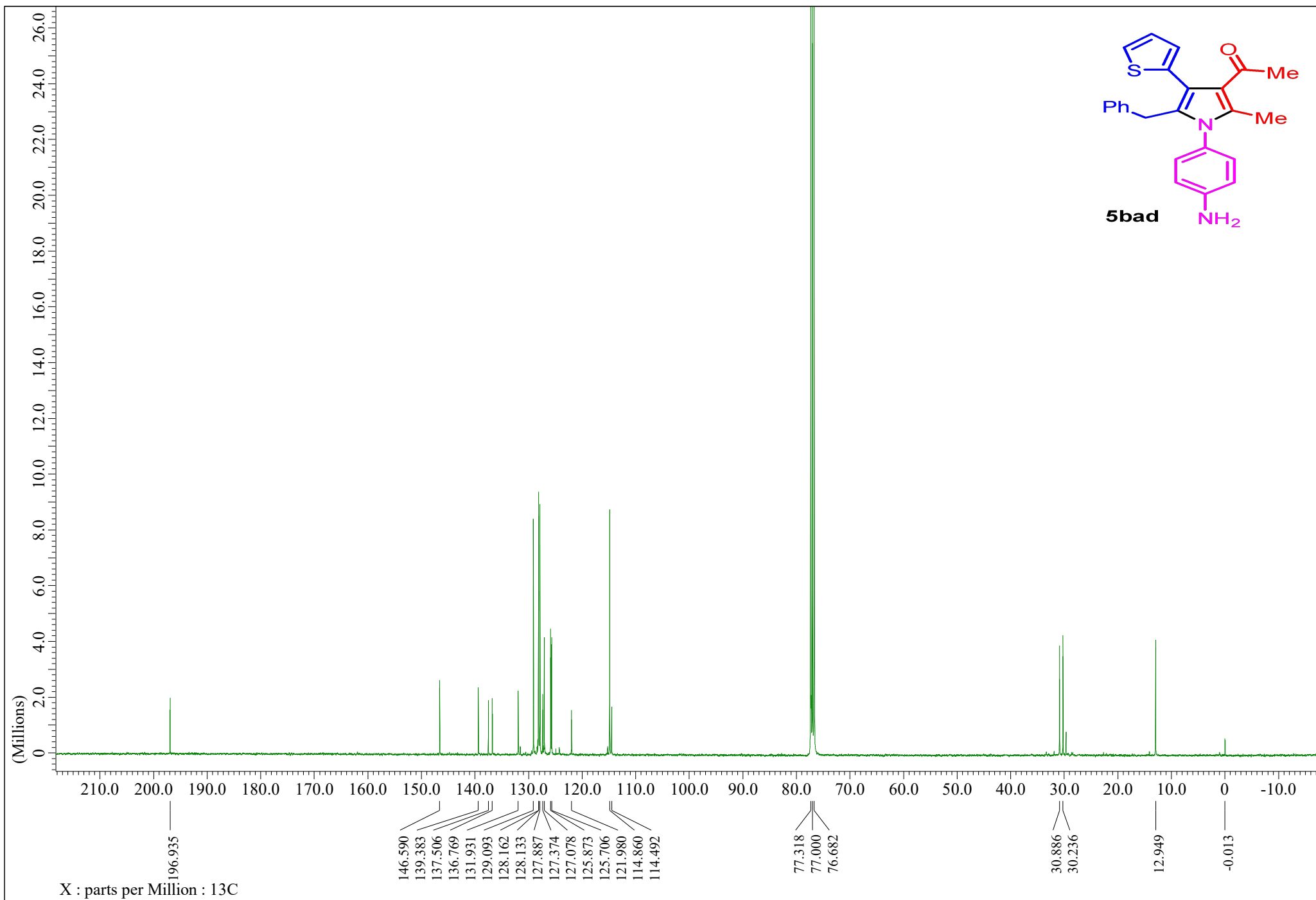

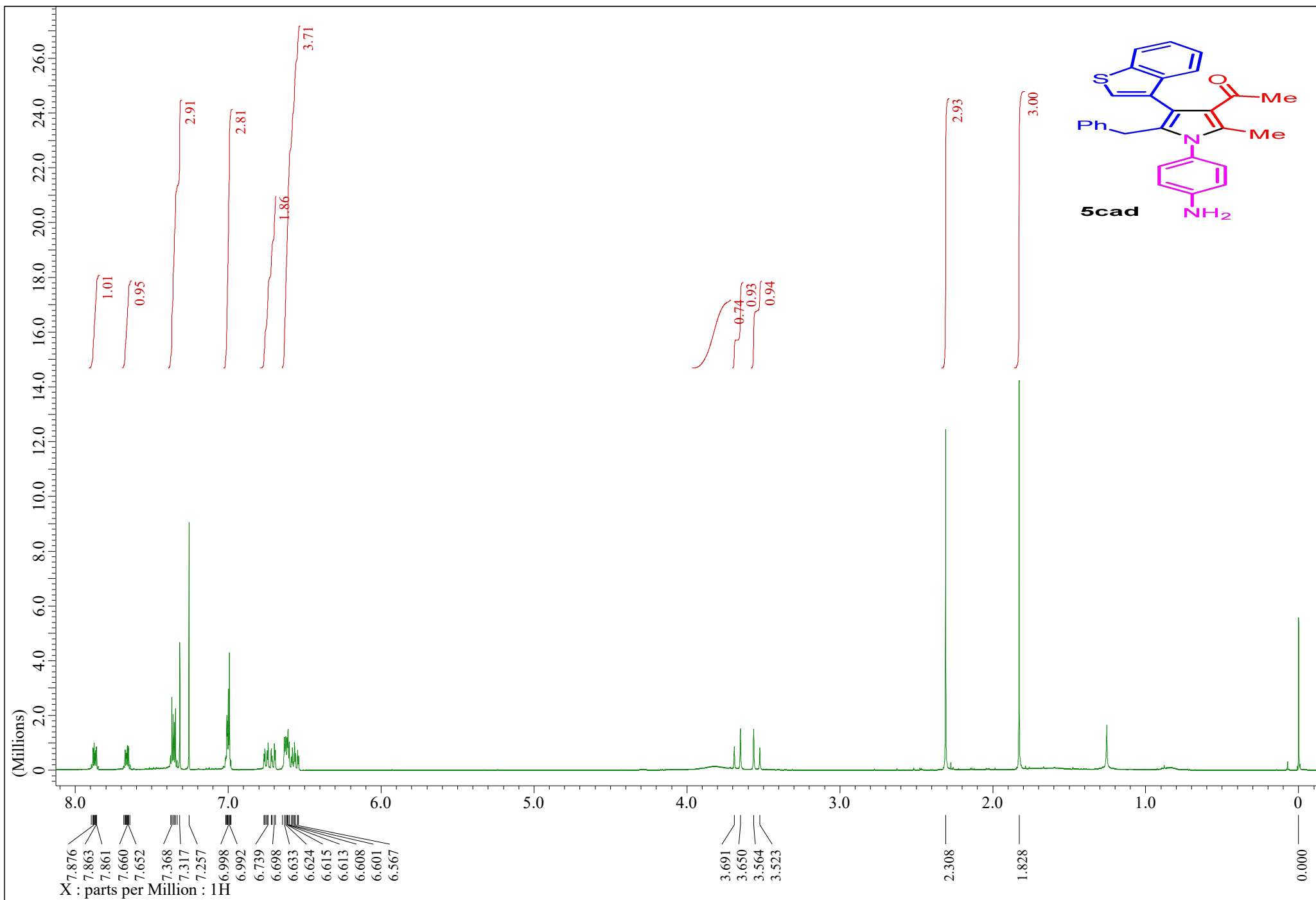

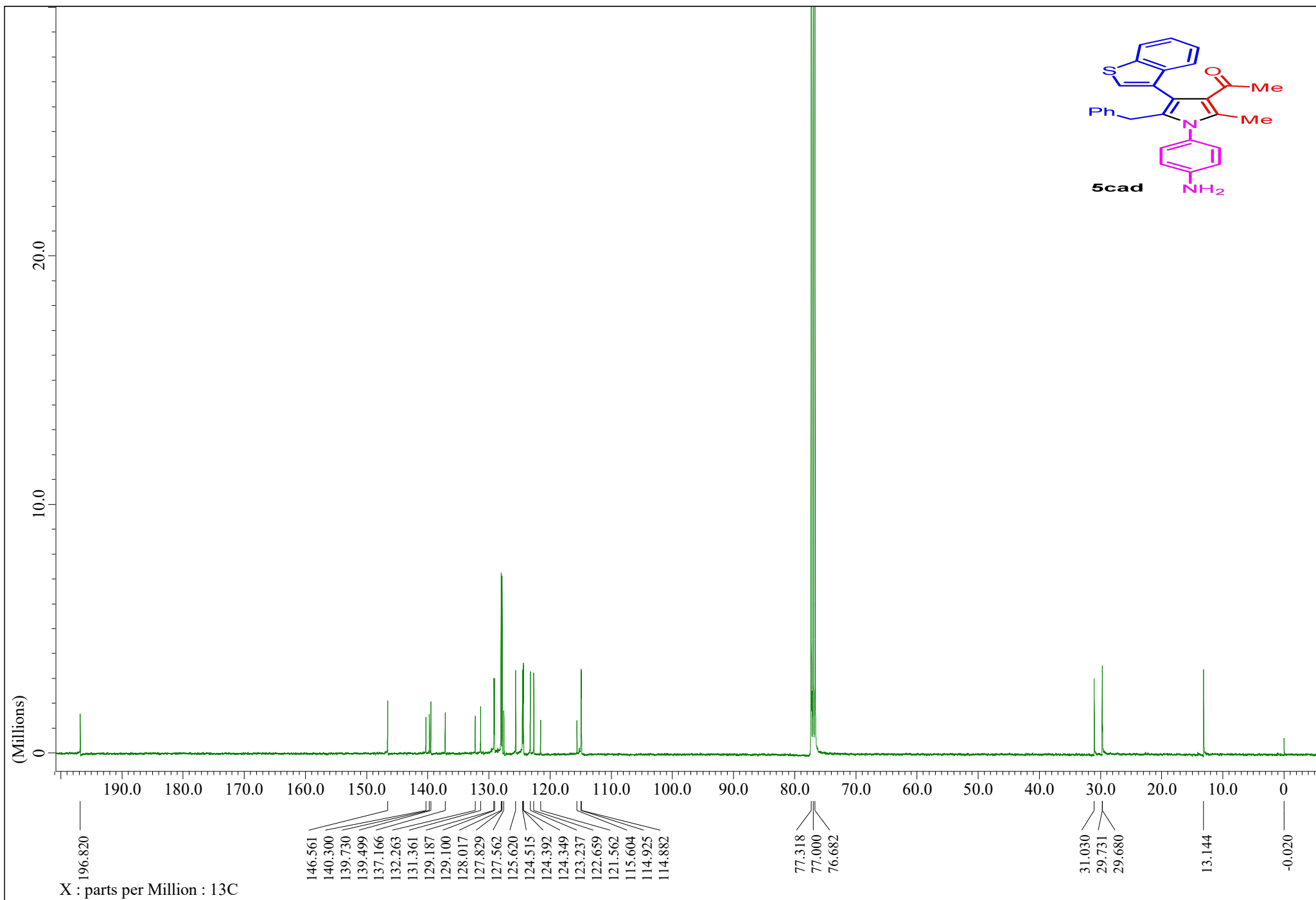

Supplement: Supplementary file 1 [file molecules-31-01203-s001.zip › molecules-4175917-supplementary.pdf]
